# METTL1-driven epitranscriptomic enhancement of TXNDC12 boosts c-Myc stability through USP5 in HNSCC

**DOI:** 10.1038/s12276-025-01512-2

**Published:** 2025-08-01

**Authors:** Zizhao Mai, Jiarong Zheng, Ye Lu, Pei Lin, Yunfan Lin, Yucheng Zheng, Xu Chen, Bing Guo, Li Cui, Xinyuan Zhao

**Affiliations:** 1 Stomatological Hospital, School of Stomatology, Southern Medical University, Guangzhou, China; 2 Department of Dentistry, The First Affiliated Hospital, Sun Yat-Sen University, Guangzhou, China; 3 School of Dentistry, University of California, Los Angeles, Los Angeles, CA USA

**Keywords:** Oral cancer, Cancer genomics

## Abstract

Head and neck squamous cell carcinoma (HNSCC) is a prevalent malignancy worldwide. Advancing understanding of the molecular mechanisms driving tumor progression and resistance to therapy is essential for developing new strategies to improve patient outcomes in HNSCC. Here we demonstrate that elevated expression of thioredoxin domain-containing protein 12 (TXNDC12) in HNSCC is associated with adverse clinical outcomes and reduced survival. Modulating TXNDC12 levels demonstrates that its reduction curtails aggressive tumor phenotypes and cisplatin resistance, while its overexpression exacerbates these characteristics. Comprehensive RNA transcriptomic analysis reveals that depletion of TXNDC12 leads to significant suppression of c-Myc signaling pathways. Mechanistically, TXNDC12 stabilizes c-Myc protein by promoting its interaction with USP5, thus preventing proteasomal degradation of c-Myc. Moreover, METTL1 enhances *TXNDC12* mRNA stability via an m^7^G-dependent mechanism. Clinical validation underscores the importance of the METTL1-TXNDC12-c-Myc axis in HNSCC. Our findings reveal that METTL1-coupled epitranscriptomic upregulation of TXNDC12 in HNSCC enhances c-Myc signaling by promoting its USP5-mediated stability.

## TXNDC12 drives aggressive HNSCC and therapy resistance

Head and neck cancers are common worldwide, with most being a type called head and neck squamous cell carcinoma (HNSCC). Researchers are looking for new ways to tackle this problem. A recent study found that a protein called thioredoxin domain-containing protein 12, TXNDC12, is often present at high levels in HNSCC tumors. This protein seems to make the cancer more aggressive and resistant to treatment. Researchers used lab experiments and animal models to study TXNDC12. They found that reducing TXNDC12 levels in cancer cells made them less aggressive and more sensitive to a chemotherapy drug called cisplatin. They also discovered that TXNDC12 helps stabilize another protein, c-Myc, which is known to promote cancer growth. The study suggests that targeting TXNDC12 could be a new way to treat HNSCC, potentially making existing treatments such as cisplatin more effective.

This summary was initially drafted using artificial intelligence, then revised and fact-checked by the author.

## Introduction

Head and neck tumors rank as the sixth most prevalent cancer globally, with head and neck squamous cell carcinoma (HNSCC) comprising over 90% of these malignancies^1,2^. HNSCC is a heterogeneous tumor originating from the squamous epithelial cells of the oral cavity, oropharynx, larynx and hypopharynx^3^. The primary risk factors for HNSCC include tobacco and alcohol consumption, human papillomavirus infection, genetic predisposition and dietary habits^4^. Despite significant advances in surgical techniques, radiotherapy, chemotherapy, molecular targeted therapy and immunotherapy, the 5-year survival rate for patients with HNSCC has not improved over the past few decades^5–7^. This stagnation is largely due to lymph node metastasis, local recurrence and high rates of treatment resistance^8–10^. Thus, there is an urgent need to identify novel molecular targets and uncover the underlying molecular mechanisms to improve the prognosis for patients with HNSCC^11^.

Thioredoxin domain-containing protein 12 (TXNDC12) is a member of the thioredoxin family of proteins, which play a crucial role in maintaining normal cellular function^12^. Recent studies have highlighted the significance of TXNDC12 in various cellular processes, including regulating ferroptosis, apoptosis and protein quality control. For instance, TXNDC12 inhibits ferroptosis by preventing lipid peroxidation independently of GPX4. Its upregulation by ATF4 confers resistance to ferroptosis, while its absence enhances ferroptosis-induced tumor suppression^13^. Interestingly, TXNDC12 ensures the proper endoplasmic reticulum quality control of ATF6α, facilitating its movement to the Golgi apparatus and subsequent processing into an active transcription factor^14^. Importantly, aberrant expression of TXNDC12 has been associated with tumor progression and poor prognosis in several malignancies^15–17^. TXNDC12 is upregulated in metastatic hepatocellular carcinoma, promoting metastasis through ZEB1-mediated epithelial–mesenchymal transition. It facilitates nuclear translocation and activation of β-catenin, which is essential for ZEB1 expression^18^. In addition, TXNDC12 modulates ferroptosis in glioma by regulating SLC7A11 expression. Knockdown of TXNDC12 enhances erastin-induced reactive oxygen species, lipid peroxidation and iron accumulation, subsequently reducing glioma cell viability^19^. Bioinformatic analyses of in-house RNA sequencing data and public databases reveal significant overexpression of *TXNDC12* in HNSCC tissues, correlating with decreased overall survival. Therefore, elucidating the role and regulatory mechanisms of TXNDC12 could provide critical insights into the molecular basis of tumorigenesis and identify potential therapeutic targets to improve patient outcomes in HNSCC.

In this study, TXNDC12 was found to be notably overexpressed in HNSCC, with higher levels correlating with aggressive clinicopathological features and reduced overall survival. Consistent findings from both in vitro and in vivo experiments demonstrated that depletion of TXNDC12 diminished malignant phenotypes and reduced cisplatin resistance in HNSCC cells. Conversely, overexpression of TXNDC12 exacerbated these traits. Unbiased RNA transcriptomic analysis indicated significant inhibition of c-Myc-related signaling pathways following TXNDC12 depletion. Further mechanistic insights revealed that TXNDC12 stabilizes c-Myc protein by enhancing the interaction between USP5 and c-Myc, thereby preventing c-Myc’s proteasome-dependent degradation. Crucially, METTL1 was shown to enhance *TXNDC12* mRNA stability via an m^7^G-dependent mechanism. The clinical relevance of the METTL1-TXNDC12-c-Myc axis was substantiated in HNSCC specimens, underlining the potential of targeting this pathway as a therapeutic strategy in HNSCC. This study underscores the pivotal role of TXNDC12 in modulating tumor biology and chemoresistance, positioning the METTL1-TXNDC12-c-Myc axis as a promising target for improving patient outcomes in HNSCC.

## Materials and methods

### Patients and clinical samples

This study was approved by the Institutional Review Board of the First Affiliated Hospital of Sun Yat-sen University and conducted in compliance with the ethical standards of the Declaration of Helsinki. Informed consent was obtained from all participants. Clinicopathological parameters such as age, gender, tumor location, smoking history, TNM stage and differentiation status were collected from electronic health records.

### Cell culture

The HNSCC cell lines UMSCC-1 (SCC-1) and UTSCC-23 (SCC-23) were obtained from the University of Michigan. These cells were cultured in Dulbecco’s modified Eagle’s medium (DMEM) with high glucose, supplemented with 10% fetal bovine serum and 1% penicillin–streptomycin. The cultures were maintained at 37 °C in a humidified atmosphere with 5% CO_2_. Routine cell culture procedures were followed to ensure optimal growth conditions.

### Plasmid cloning, lentivirus generation and siRNA/plasmid transfection

Short hairpin RNA (shRNA) oligonucleotides targeting TXNDC12 were cloned into the LV3-pGLV-h1-GFP-puro vector. Meanwhile, the full-length TXNDC12 gene was cloned into the pGCL–GFP lentiviral vector. To generate lentiviral particles, HEK293T cells were cotransfected with these recombinant vectors along with packaging plasmids. After 72 h of incubation, the supernatants containing the lentiviruses were collected and concentrated. The HNSCC cells were infected with these lentiviruses at a multiplicity of infection of 30. In a similar fashion, lentiviral particles for METTL1 overexpression or shRNA targeting c-Myc were produced. For transient knockdown experiments, small interfering RNAs (siRNAs) targeting USP5 and METTL1 were synthesized by RiboBio and introduced into the cells using Lipofectamine RNAiMAX (Thermo Fisher Scientific). Plasmid DNA transfections were performed using Lipofectamine 3000 (Thermo Fisher Scientific) as per the manufacturer’s protocol. The sequences of the oligonucleotides used in this study are provided in Supplementary Table 1.

### MTT assay

The cells were seeded at a density of 3000 per well in 96-well plates following the indicated genetic modifications. Post seeding, the plates were incubated for periods ranging from 1 to 4 days. At specified intervals, 20 μl of 3-(4,5-dimethylthiazol-2-yl)-2,5-diphenyltetrazolium bromide (MTT) solution (5 mg ml^−1^) was introduced into each well. The plates were then returned to a 37 °C incubator with 5% CO_2_ for a further 4-h incubation. After this, the medium was removed, and 200 μl of dimethyl sulfoxide was added to solubilize the resulting formazan. Absorbance readings at 570 nm were taken using a Synergy HT multidetection reader (Bio-Tek Instruments).

### Colony formation assay

The cells subjected to specific treatments were seeded into six-well plates and allowed to incubate for 2 weeks to facilitate colony formation. After the incubation period, the cells were fixed with 4% paraformaldehyde for 15 min at room temperature. The fixed cells were then stained with a 0.5% crystal violet solution for 30 min. Excess stain was washed away with distilled water, and the plates were left to air dry before analysis.

### EdU assay

The cells were incubated with a 10 μmol l^−1^ 5-ethynyl-2'-deoxyuridine (EdU) solution at 37 °C for 2 h, followed by fixation with 4% paraformaldehyde and permeabilization using 0.5% Triton X-100. They were then exposed to a Click-iT Plus reaction cocktail (Invitrogen) in a dark environment at room temperature for 30 min. After a PBS wash, the nuclei were stained with Hoechst 33342, and fluorescence microscopy images were obtained with a Leica Microsystems inverted microscope.

### Matrigel invasion assay

The invasion capacity of cancer cells was assessed using chambers coated with Matrigel from BD Biosciences. A suspension of cancer cells at a density of 5.0 × 10^5^ cells in 300 µl of DMEM was seeded into the upper part of each Transwell chamber, while the bottom chamber was filled with DMEM enriched with 10% fetal bovine serum. After incubating for 24 h, the cells remaining on the upper side of the membrane were carefully wiped away. The cells that had migrated through the membrane were fixed with 4% paraformaldehyde, stained with 0.5% crystal violet and analyzed. The cell invasion was quantified by calculating the average area of invasion using Image software.

### Kinetic would healing assay

The cells were seeded into 96-well plates (Essen ImageLock, Essen Instruments) and a wound was created using a wound scratcher (Essen Instruments). Wound confluence was monitored using the Incucyte Live-Cell Imaging System and software (Essen Instruments). Wound closure was observed every 2 h for 48 h, with the mean relative wound density compared across at least five biological replicates per experiment.

### Quantitative PCR

The total RNA was extracted from cellular samples using a Quick-RNA extraction kit (Zymo Research Corp) according to the provided protocol. Complementary DNA synthesis was performed using SuperScript III reverse transcriptase (Invitrogen). The synthesized cDNA was then amplified using SYBR Green I MasterMix (Roche, Applied Sciences) and quantified on a LightCycler 96 Instrument (Roche). Relative gene expression was analyzed using the 2^−ΔΔCT^ method, with GAPDH serving as the normalization control. Details of the primers used are listed in Supplementary Table 1.

### Western blot

Protein lysates were separated on 4–20% SDS–polyacrylamide gels and then transferred to 0.2 µm PVDF membranes using a Trans-Blot Turbo system (Bio-Rad). The membranes were blocked for 10 min at room temperature using blocking buffer (EpiZyme) and incubated overnight at 4 °C with primary antibodies, followed by five, 5-min washes with TBST. They were then treated with HRP-conjugated secondary antibodies (Proteintech) for an hour at room temperature. The protein detection was conducted using Amersham ECL Prime Western Blotting Detection Reagent (Cytiva). The primary antibodies used in this study, all sourced from Proteintech, included: TXNDC12, c-Myc, METTL1, USP5, GAPDH and epitope tag antibodies.

### Co-IP assay

The cells undergoing specified treatments were lysed using RIPA buffer, followed by centrifugation at 14,000*g* for 20 min at 4 °C to obtain the supernatant. The supernatant was then incubated with primary antibodies overnight at 4 °C. To capture the immune complexes, the lysate–antibody mixture was added to prewashed magnetic beads (EpiZyme) and incubated for 6 hours at 4 °C, allowing the formation of antigen–antibody–bead complexes. These complexes were washed thrice with elution buffer, denatured by heating at 100 °C for 10 min and finally analyzed by western blot to assess protein interactions.

### Liquid chromatography with tandem mass spectrometry

Proteins interacting with TXNDC12 were separated using SDS–polyacrylamide gels. The selected bands were then excised, digested enzymatically with sequencing-grade trypsin (Promega) and subjected to tandem tandem mass spectrometry for protein identification.

### CHX chase assay

The cells with indicated treatments were exposed to 100 μg ml^−1^ cycloheximide (CHX), an inhibitor of protein biosynthesis, to halt the synthesis of new proteins. Following this treatment, the protein lysates were systematically collected at specified time intervals. These samples were then processed through western blot analysis to quantify degradation kinetics of c-Myc, enabling a detailed assessment of its stability under protein synthesis inhibition conditions.

### RNA stability analysis

The cells subjected to the indicated treatments were exposed to actinomycin D (5 μg ml^−1^) for 0, 2, 4 and 8 h. The total RNA was extracted at each time point, and the dynamic expression levels of *TXNDC12* mRNA were measured using quantitative PCR.

### m^7^G RNA immunoprecipitation assay

The m^7^G RNA immunoprecipitation assay was performed using the Magna RIP Kit (Millipore) according to the manufacturer’s instructions. For immunoprecipitation, m^7^G antibody was employed to isolate m^7^G-modified mRNA. The isolated mRNA was subsequently analyzed by quantitative PCR. The fold enrichment was calculated by normalizing the data to the input RNA.

### IHC

Formalin-fixed, paraffin-embedded tissue specimens underwent xylene deparaffinization and were sequentially rehydrated in decreasing concentrations of ethanol. Following rehydration, the sections were blocked using goat serum to prevent nonspecific binding and then incubated with primary antibodies at 4 °C throughout the night. Subsequently, the sections were washed three times with phosphate-buffered saline and exposed to a horseradish peroxidase-conjugated secondary antibody for 1 h at room temperature. Visualization of the antigen–antibody interaction was achieved using a diaminobenzidine staining kit. For quantitative analysis, the *H*-score for each slide was calculated using a weighted sum that accurately reflects both the intensity and the proportion of cells displaying each level of staining. The formula for the *H*-score is: *H*-score = (3 × percent of cells with strong staining) + (2 × percent of cells with strong staining) + (1 × percent of cells with strong staining). The cells that showed no staining were not included in the calculation. This scoring process was independently conducted by two pathologists who were not privy to the clinical data associated with the samples, ensuring unbiased assessment.

### Animal experiments

Animal studies were conducted in strict adherence to guidelines approved by the Institutional Animal Care and Ethics Committee at Southern Medical University. The mice used in the experiments were procured from the Guangdong Medical Laboratory Animal Center (Foshan). For the carcinogenesis model, 6-week-old C57BL/6 mice were administered 4-nitroquinoline 1-oxide at a concentration of 50 μg ml^−1^ in their drinking water for 16 weeks, followed by a subsequent 8–10 weeks on normal water to allow tumor development. In addition, to establish a xenograft model, 6-week-old BALB/c nude mice were subcutaneously injected in the dorsal flank with 2 × 10^6^ cells that had undergone specific treatments. The health status and tumor growth of these mice were rigorously monitored throughout the study. A total of 6 weeks post injection, the mice were euthanized, and the tumors were surgically removed to measure volume and weight. The excised tumors were then fixed, embedded in paraffin and prepared for immunohistochemical analysis to assess the expression of Ki-67.

### RNA sequencing

RNA sequencing was conducted on the NovaSeq 6000 platform at Majorbio Bio-Pharm Technology. The libraries were prepared using the Illumina TruSeq RNA Sample Preparation Kit (Illumina) according to the manufacturer’s guidelines. Raw paired-end reads were trimmed and quality-checked using SeqPrep and Sickle with default settings. The reads were aligned to the reference genome with the HISAT pipeline and assembled into transcripts using StringTie. Differentially expressed genes were identified using DEGseq, with criteria set at an absolute fold change ≥2 and an adjusted *P* value ≤0.05.

### Bioinformatic analysis

Datasets GSE10121, GSE25099, GSE25727, GSE26549, GSE30784, GSE31056, GSE37991, GSE40774, GSE47443, GSE55550, GSE41613, GSE58911, GSE85195, GSE85446, GSE127165 and GSE143224 were downloaded from the National Center for Biotechnology Information Gene Expression Omnibus database (https://www.ncbi.nlm.nih.gov/geo/). For the TCGA HNSCC cohort, RNA-seq data and corresponding clinical information were obtained from the National Cancer Institute Genomic Data Commons portal (https://gdc.cancer.gov/). Using X-tile software (https://medicine.yale.edu/lab/rimm/research/software/), the optimal cutoff value was determined to categorize patients with HNSCC into high and low *TXNDC12* expression groups. Subsequent survival analysis was then performed. A gene set enrichment analysis (GSEA) was conducted to investigate how differences in *TXNDC12* expression affect cellular pathways. The patients were categorized into groups with high and low *TXNDC12* expression based on the median value of *TXNDC12*. A GSEA then systematically identified which pathways and gene sets were significantly upregulated or downregulated in each group.

### Statistical analysis

Statistical analysis was executed utilizing GraphPad Prism version 9.0 (GraphPad Software). Group comparisons were performed through one-way analysis of variance and Student’s *t*-tests, with findings presented as means ± standard deviations, unless specified otherwise. Survival probabilities were estimated using the Kaplan–Meier method, and the differences between survival curves were analyzed using the log-rank test. The Pearson correlation coefficient was used to quantify the association between two variables. Statistical significance was set at *P* values <0.05.

## Results

### TXNDC12 overexpression is associated with unfavorable clinical outcome in HNSCC

**Fig. 1:**
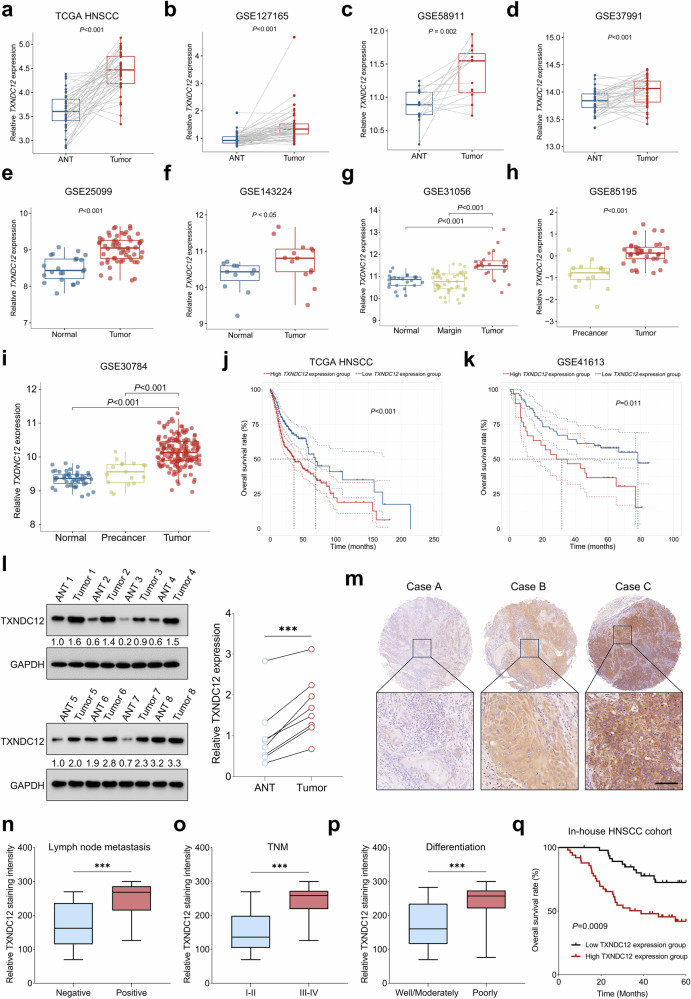
TXNDC12 overexpression is associated with unfavorable clinical outcome in HNSCC. **a**–**d** *TXNDC12* levels in tumor versus ANT across TCGA HNSCC (**a**), GSE127165 (**b**), GSE58911 (**c**) and GSE37991 (**d**) datasets. **e**–**g** Differential expression of *TXNDC12* in tumor compared with normal tissues across GSE25099 (**e**), GSE143224 (**f**) and GSE31056 (**g**) datasets. **h**, **i** The levels of *TXNDC12* across normal, precancerous and tumor tissues in GSE85195 (**h**) and GSE30784 (**i**) datasets. **j**, **k** Survival analysis stratifying HNSCC patients by *TXNDC12* expression using TCGA HNSCC cohort (normalized *TXNDC12* expression cutoff value of 4.427) (**j**) and GSE41613 dataset (normalized *TXNDC12* expression cutoff value of 10.405) (**k**). **l** A western blot analysis comparing TXNDC12 levels in eight pairs of tumor tissues (*n* = 8) and ANTs (*n* = 8). **m** Representative images showing low, medium and strong TXNDC12 staining intensities in tissue sections from an in-house HNSCC cohort (*n* = 97). **n**–**p** An analysis of TXNDC12 staining intensity in patients with HNSCC, stratified by clinical parameters such as lymph node metastasis (**n**), TNM stage (**o**) and tumor grade (**p**). **q** Survival curves based on TXNDC12 expression levels in the in-house HNSCC cohort (*H*-score cutoff value of 192.15). Statistical significance was assessed by the log-rank test.

To elucidate the potential clinical significance of *TXNDC12* in HNSCC, we first assessed the differential expression patterns of *TXNDC12* among HNSCC tissues, adjacent normal tissues (ANTs) and normal tissues, utilizing publicly accessible databases. Our analysis demonstrated a significant upregulation of *TXNDC12* expression in HNSCC tissues compared with paired ANTs across multiple cohorts, including data from TCGA HNSCC cohort, as well as GSE127165, GSE58911 and GSE37991 (Fig. 1a–d). This trend was consistently observed when comparing malignant tissues with normal tissues in additional datasets, such as GSE25099, GSE143224 and GSE30156 (Fig. 1e–g). Notably, *TXNDC12* expression levels were significantly elevated in tumor tissues relative to precancerous lesions in the GSE85195 and GSE30784 datasets (Fig. 1h, i). Survival analysis further demonstrated that patients with HNSCC exhibiting high *TXNDC12* expression levels experienced significantly poorer overall survival compared with those with low expression across independent cohorts (Fig. 1j, k). The potential prognostic value of *TXNDC12* was also assessed in other cancer types using data from TCGA. This analysis revealed that high *TXNDC12* expression consistently correlated with worse overall survival in patients with cervical squamous cell carcinoma, esophageal carcinoma, acute myeloid leukemia, lower grade glioma, liver hepatocellular carcinoma, kidney renal clear cell carcinoma, kidney renal papillary cell carcinoma, mesothelioma, sarcoma, thyroid carcinoma and uveal melanoma (Supplementary Fig. 1).

The bioinformatic analyses presented suggest that TXNDC12 may play a crucial role in the pathogenesis of HNSCC, underscoring the need for further investigation into its potential as a therapeutic target. Consistent with data from public databases, TXNDC12 protein expression was notably upregulated in HNSCC tissues compared with ANTs (Fig. 1l). Moreover, we compared the expression patterns of TXNDC12 in normal mouse tongue tissues and tumor tissues from a 4-nitroquinoline 1-oxide-induced oral cancer model. The results indicated a significant increase in TXNDC12 expression in tumor tissues (Supplementary Fig. 2a, b). Immunohistochemistry (IHC) staining of our in-house HNSCC cohort revealed that TXNDC12 staining intensity was significantly higher in patients with lymph node metastasis, advanced TNM stage or poorly differentiated carcinoma compared with their corresponding controls (Fig. 1m–p and Supplementary Table 2). Furthermore, patients with HNSCC in the high TXNDC12 expression group exhibited significantly worse overall survival compared with those in the low expression group (Fig. 1q). In early-stage HNSCC (TNM stage I–II), patients with high TXNDC12 expression exhibited markedly reduced overall survival compared with those with low expression (Supplementary Fig. 3a). Although a similar trend was observed in advanced-stage patients (TNM stage III–IV), the difference did not reach statistical significance (Supplementary Fig. 3b). When comparing overall survival between oral cavity and nonoral cavity tumors irrespective of TXNDC12 expression, no significant difference was found, indicating that tumor location alone does not account for the observed prognostic variation (Supplementary Fig. 3c). However, stratified analyses within these anatomical subgroups demonstrated that high TXNDC12 expression was significantly associated with worse survival in both nonoral cavity and oral cavity tumors (Supplementary Fig. 3d,e). These results underscore the correlation between high TXNDC12 expression and aggressive disease features, as well as adverse clinical outcomes in HNSCC.

### TXNDC12 enhances the malignant characteristics of HNSCC cells

**Fig. 2:**
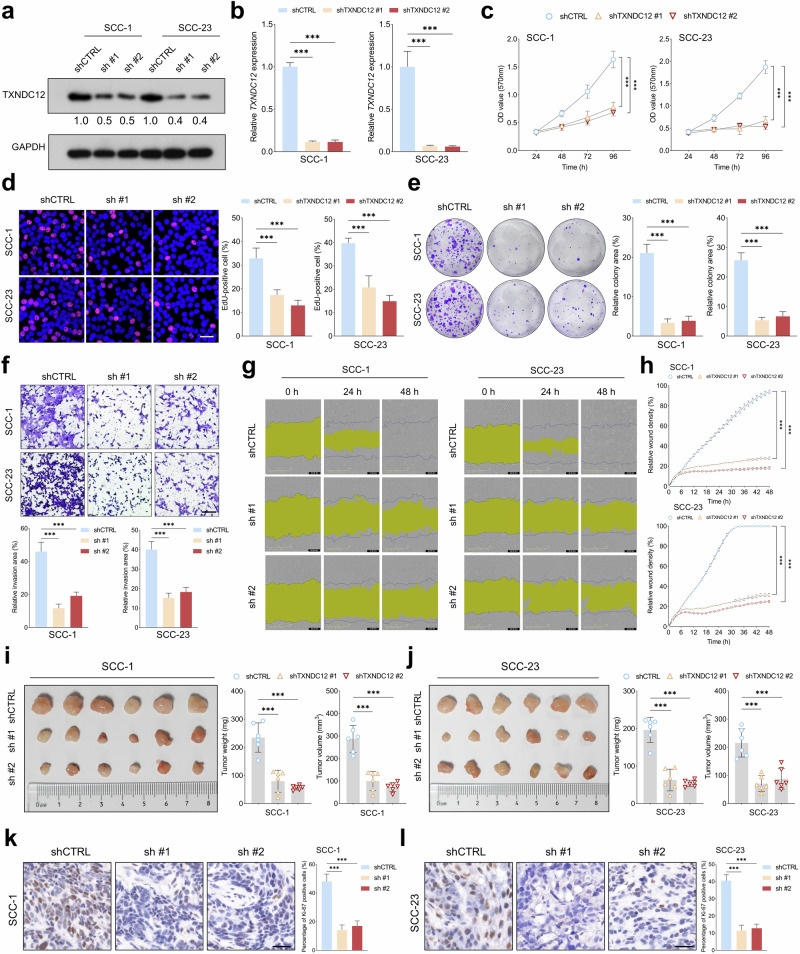
TXNDC12 depletion suppressed the malignant phenotypes of HNSCC cell. **a**, **b** Western blot (*n* = 3 biologically independent samples) (**a**) and quantitative PCR analyses (*n* = 3 biologically independent experiments, each with three technical replicates) (**b**) were performed to assess TXNDC12 expression in HNSCC cells following transfection with shTXNDC12 or shCTRL (control group for shTXNDC12). **c** MTT assay results, measured as optical density, demonstrated differences in cell proliferation over time between shTXNDC12-treated and shCTRL-treated cells (*n* = 3 biologically independent experiments, each with five technical replicates). **d** The EdU incorporation assay quantified the proportion of EdU positive cells, comparing TXNDC12-depleted cells with controls (*n* = 4 biologically independent samples). **e** Colony formation assay evaluated the clonogenic potential of HNSCC cells with or without TXNDC12 depletion (scale bar, 50 μm) (*n* = 4 biologically independent samples). **f** The Transwell invasion assay assessed the invasive potential of TXNDC12-depleted cells compared with the control counterpart (scale bar, 200 μm) (*n* = 4 biologically independent samples). **g**, **h** Wound healing assays were conducted to measure migration differences between TXNDC12-depleted (**g**) and control cells (**h**) (*n* = 4 biologically independent samples). **i**, **j** Xenograft experiments quantified tumor growth by comparing tumor volume and weight between TXNDC12-depleted groups (*n* = 6) (**i**) and the control group (*n* = 6) (**j**). **k**, **l** Immunohistochemical analysis of Ki-67 labeling indexed the proliferation rates in xenograft tumors derived from TXNDC12-depleted (**k**) and control cells (**l**) (scale bar, 100 μm).

To elucidate the impact of TXNDC12 depletion on the malignant behaviors of HNSCC cells, stable TXNDC12 knockdown cell lines were established (Fig. 2a, b). MTT assays indicated a significant reduction in OD in TXNDC12-depleted cells compared with controls (Fig. 2c). This finding was complemented by EdU incorporation assays, which showed a reduced proportion of cells in the DNA synthesis phase post-TXNDC12 knockdown (Fig. 2d). The colony formation assay further corroborated these results, revealing a pronounced decrease in colony formation ability of HNSCC cells upon TXNDC12 depletion (Fig. 2e). In assessments of cell motility, Transwell invasion assays demonstrated a significant reduction in the invasive capabilities of the cells with downregulated TXNDC12 (Fig. 2f), and wound healing assays confirmed a marked suppression in cell migration in the TXNDC12-depleted group compared with controls (Fig. 2g, h). Notably, in a nude mouse subcutaneous tumor model, there were significant reductions in both tumor volume and weight in the group with TXNDC12 depletion compared with the control group (Fig. 2i, j). Furthermore, IHC analysis of tumor sections revealed a lower proportion of Ki-67-positive cells in tumors formed from HNSCC cells with TXNDC12 depletion, indicating reduced proliferative activity (Fig. 2k, l).

To investigate the role of TXNDC12 overexpression in promoting malignant behaviors in HNSCC cells, stable cell lines with enforced expression of TXNDC12 were developed (Supplementary Fig. 4a,b). MTT, EdU and colony formation assays consistently showed that TXNDC12 overexpression significantly increased both proliferation and colony formation capabilities of HNSCC cells (Supplementary Fig. 4c–e). In addition, TXNDC12 overexpression markedly enhanced the invasive ability of these cells, as demonstrated in transwell invasion assay (Supplementary Fig. 4f). In vivo experiments demonstrated that TXNDC12 overexpression significantly enhanced the tumorigenic capacity of HNSCC cells, as evidenced by increased tumor volume and weight in a nude mouse model (Supplementary Fig. 4g–j). Furthermore, elevated Ki-67 staining in sections from TXNDC12-overexpressing tumors confirmed a higher rate of cellular proliferation (Supplementary Fig. 4k). These results collectively underscore the contribution of TXNDC12 overexpression to the aggressive phenotype and enhanced tumorigenic potential in HNSCC.

### TXNDC12 modulates the sensitivity of HNSCC cells to cisplatin in vitro and in vivo

**Fig. 3:**
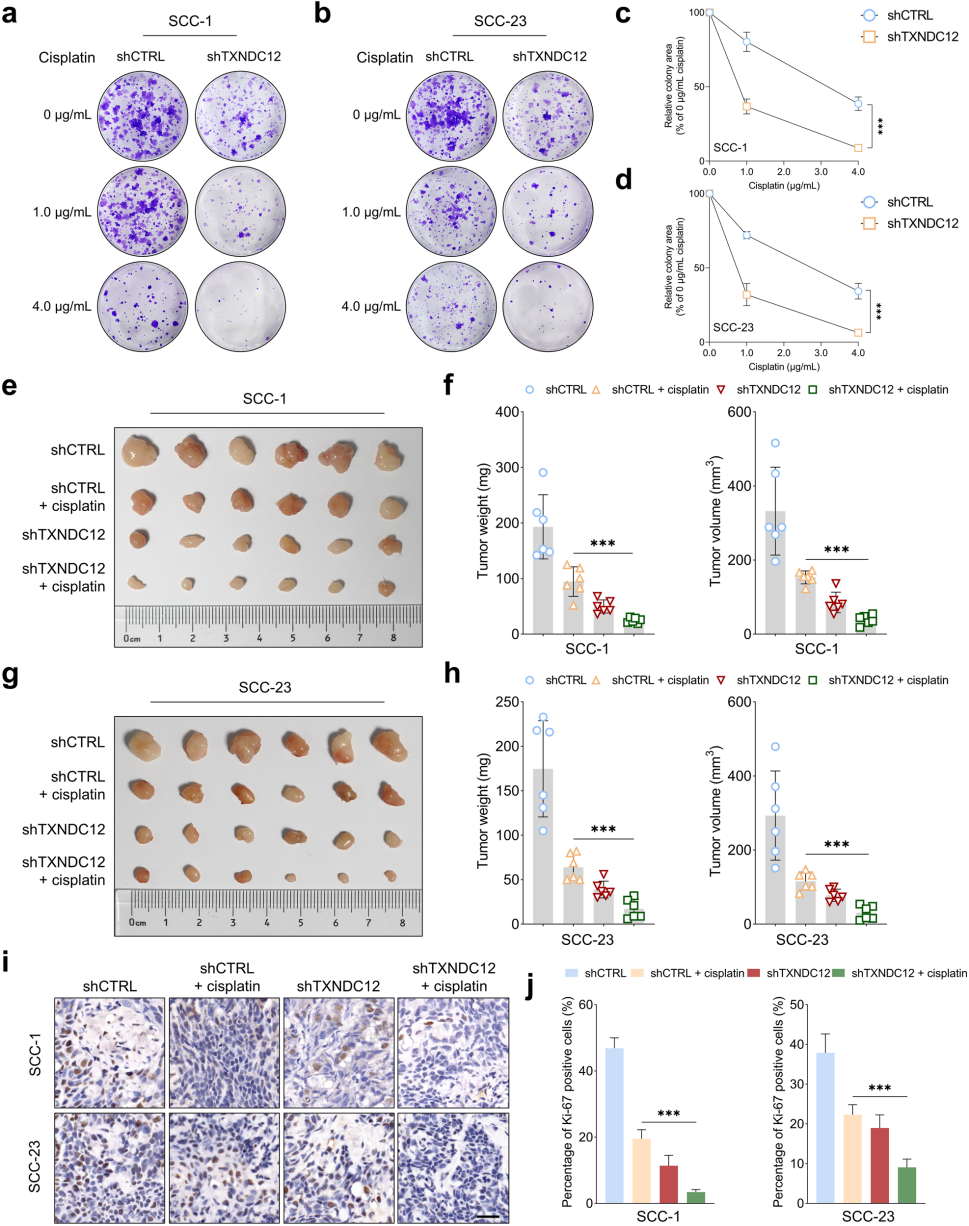
TXNDC12 depletion enhances the inhibitory effect of cisplatin in HNSCC cells. **a**–**d** Assessment of colony formation capacity in SCC-1 (**a, c**) and SCC-23 (**b, d**) cells subjected to various treatments (*n* = 4 biologically independent samples). **e**–**h** In a nude mouse subcutaneous model, SCC-1 (**e, f**) or SCC-23 (**g, h**) cells (1 × 10^6^ cells per mouse) were injected subcutaneously to establish tumors. Tumor growth was quantitatively evaluated by comparing tumor volume and weight across four groups: shCTRL (control group for shTXNDC12) (*n* = 6), shCTRL + cisplatin-treated (5 mg kg^−1^, intraperitoneally every 5 days) (*n* = 6), shTXNDC12-treated (*n* = 6) and combined cisplatin + shTXNDC12 treatment (*n* = 6). **i**, **j** Immunohistochemical staining (**i**) and analysis (**j**) was performed to measure Ki-67 intensities in xenograft tumors derived from each treatment group (scale bar, 100 μm).

As TXNDC12 has been implicated in regulating tumor aggressiveness, we next investigated whether its depletion would potentiate the inhibitory effects of cisplatin in HNSCC cells. Colony formation assays demonstrated that combining TXNDC12 depletion with cisplatin significantly reduced the colony-forming capacity of the cancer cells more than any single treatment (Fig. 3a–d). In a subcutaneous nude mouse model, this combination treatment markedly inhibited tumor growth, showing a greater reduction in tumor volume and weight compared with the effects of either treatment alone (Fig. 3e–h). IHC analysis of tumor sections revealed a significantly lower proliferation rate, as indicated by reduced Ki-67 staining, in the combination treatment group (Fig. 3i, j). Notably, colony formation assays in SCC-1 and SCC-23 cells showed that TXNDC12 overexpression markedly increased clonogenic survival following cisplatin treatment compared with the control group. Under increasing concentrations of cisplatin, control cells exhibited a substantial reduction in colony formation, whereas TXNDC12-overexpressing cells retained significantly higher colony-forming capacity (Supplementary Fig. 5a–d). In the SCC-1 subcutaneous xenograft model, tumors derived from TXNDC12-overexpressing cells were less responsive to cisplatin treatment, as evidenced by higher tumor weight and volume in the TXNDC12-OE + cisplatin group compared with the CTRL + cisplatin group (Supplementary Fig. 5e,f). These findings suggest that TXNDC12 overexpression may reduce the sensitivity of HNSCC cells to cisplatin.

### TXNDC12 is crucial for maintaining c-Myc protein stability in HNSCC cells

**Fig. 4:**
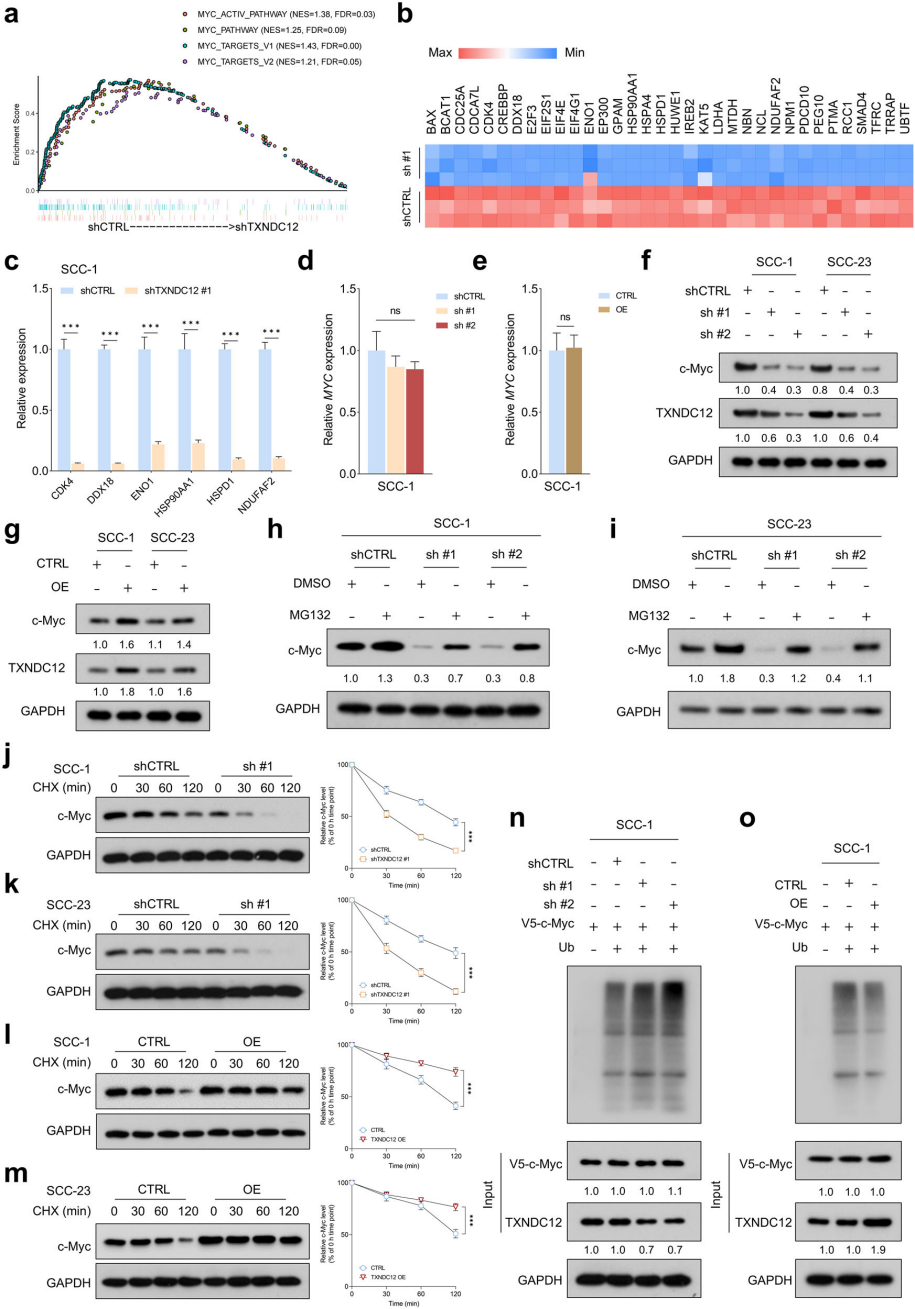
TXNDC12 is crucial for maintaining c-Myc protein stability in HNSCC cells. **a** AGSEA analysis showing the enrichment of c-Myc-related signatures in the control group compared with the TXNDC12-depleted group. **b** A heat map illustrating the expression of representative c-Myc target genes in TXNDC12-depleted and control cells. **c** A quantitative PCR analysis demonstrating the impact of TXNDC12 depletion on the expression of c-Myc target genes in SCC-1 cells (*n* = 3 biologically independent experiments, each with three technical replicates). **d**, **e** Quantitative PCR analysis showing the effects of TXNDC12 depletion (**d**) or overexpression (**e**) on MYC mRNA levels in SCC-1 cells (*n* = 3 biologically independent experiments, each with three technical replicates). **f**, **g** A western blot analysis revealing the influence of TXNDC12 depletion (**f**) or overexpression (**g**) on c-Myc protein levels in HNSCC cells (*n* = 3 biologically independent samples). **h**, **i** A western blot analysis of c-Myc protein levels in SCC-1 (**h**) and SCC-23 (**i**) cells with the indicated treatments, with or without MG132 (*n* = 3 biologically independent samples). **j**, **k** CHX chase assay illustrating the effect of TXNDC12 depletion on the degradation rate of c-Myc protein in SCC-1 (**j**) and SCC-23 (**k**) cells (*n* = 3 biologically independent samples). **l**, **m** CHX chase assay demonstrating the impact of TXNDC12 overexpression on c-Myc protein stability in SCC-1 (**l**) and SCC-23 (**m**) cells (*n* = 3 biologically independent samples). **n**, **o** An analysis of ubiquitinated c-Myc expression in SCC-1 cells subjected to the TXNDC12-depleted (**n**) or overexpressed (**o**) treatments (*n* = 3 biologically independent samples). ns, not significant.

To elucidate the potential molecular mechanisms underlying the tumor-promoting role of TXNDC12 in HNSCC, RNA sequencing was conducted to identify genes differentially expressed between TXNDC12-depleted cells and control cells. A GSEA revealed significant enrichment of c-Myc-related signatures—MYC_ACTIVE_PATHWAY (normalized enrichment score (NES) of 1.38, false discovery rate (FDR) of 0.03), MYC_PATHWAY (NES of 1.25, FDR of 0.09), MYC_TARGETS_V1 (NES of 1.43, FDR <0.01) and MYC_TARGETS_V2 (NES of 1.21, FDR of 0.05)—in the control group compared with the TXNDC12-depleted group (Fig. 4a). Interestingly, these pathways were consistently enriched in the high *TXNDC12* expression group across multiple HNSCC cohorts, including TCGA HNSCC, GSE127165, GSE40774, GSE31056, GSE30784 and GSE25727 (Supplementary Fig. 6a–f). A heat map analysis highlighted representative differentially expressed genes targeted by c-Myc between TXNDC12-depleted and control cells (Fig. 4b). A robust positive correlation between *TXNDC12* and c-Myc targeted genes was consistently observed in multiple independent HNSCC cohorts, such as TCGA HNSCC, GSE55550, GSE127165 and GSE136037 (Supplementary Fig. 7). Notably, depletion of TXNDC12 substantially reduced the expression of c-Myc targeted genes, as confirmed by quantitative PCR analysis (Fig. 4c and Supplementary Fig. 8a). This evidence collectively suggests that TXNDC12 may enhance the tumorigenic capacity in HNSCC by modulating c-Myc expression.

We subsequently assessed the impact of TXNDC12 depletion and overexpression on the expression pattern of c-Myc. Interestingly, no significant changes were observed in *MYC* mRNA levels between TXNDC12-depleted or overexpressing cells and their respective controls (Fig. 4d, e and Supplementary Fig. 8b,c). However, TXNDC12 depletion markedly reduced the protein levels of c-Myc in HNSCC cells, while overexpression of TXNDC12 led to increased c-Myc protein levels (Fig. 4f, g). Notably, treatment with the proteasome inhibitor MG132 effectively rescued the decrease in c-Myc protein levels mediated by TXNDC12 knockdown (Fig. 4h, i). This observation suggests that TXNDC12 may regulate c-Myc expression at the post-transcriptional level, potentially involving proteasomal degradation pathways. The CHX chase assay revealed that the degradation rate of c-Myc protein was significantly accelerated in TXNDC12-depleted cells compared with control cells (Fig. 4j, k). Conversely, TXNDC12 overexpression substantially decreased the rate of c-Myc degradation (Fig. 4l, m). Moreover, TXNDC12 depletion significantly elevated the ubiquitination levels of c-Myc protein, whereas overexpression of TXNDC12 produced the opposite effect, decreasing ubiquitination (Fig. 4n, o and Supplementary Fig. 8d,e). These findings underscore the critical role of TXNDC12 in modulating the ubiquitin–proteasome pathway of c-Myc degradation.

### TXNDC12 promotes tumorigenic potential of HNSCC by modulating c-Myc expression

**Fig. 5:**
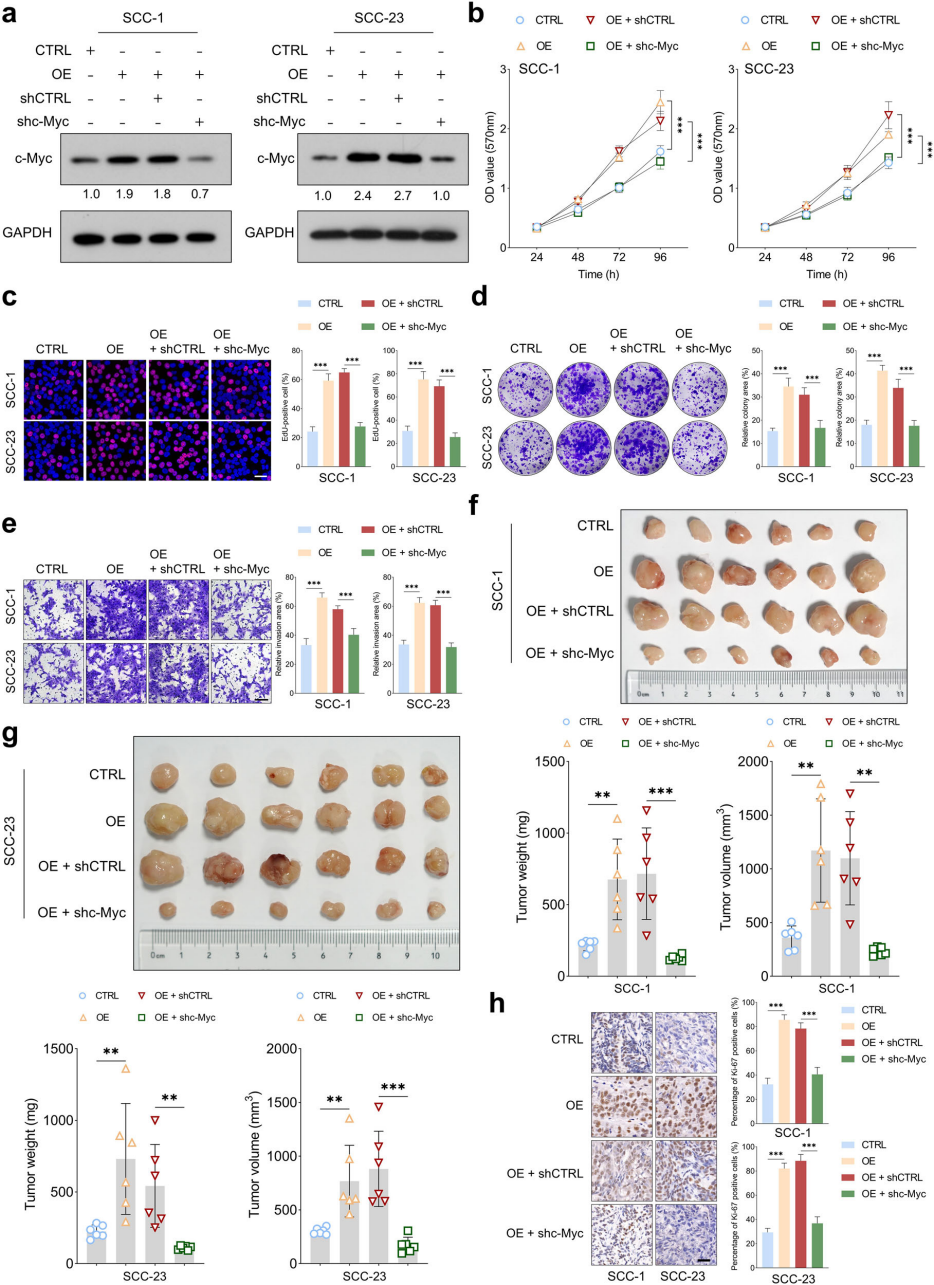
TXNDC12 promotes tumorigenic potential of HNSCC by modulating c-Myc expression. **a** A western blot analysis of c-Myc expression in HNSCC cells subjected to the specified treatments (*n* = 3 biologically independent samples). **b**–**d** MTT (*n* = 3 biologically independent experiments, each with five technical replicates) (**b**), EdU (*n* = 4 biologically independent samples) (**c**) and colony formation assays (*n* = 4 biologically independent samples) (**d**) were used to evaluate the proliferative and clonogenic potentials of HNSCC cells under the indicated treatments. **e** A Transwell invasion assay demonstrating the invasive capacity of HNSCC cells with the specified treatments (*n* = 4 biologically independent samples). **f**, **g** In a nude mouse subcutaneous model, tumor growth for SCC-1 (**f**) or SCC-23 (**g**) cells was quantitatively assessed by comparing tumor volume and weight across four groups: control (*n* = 6), TXNDC12 overexpression alone (*n* = 6), TXNDC12 overexpression with shCTRL (control group for shc-Myc) (*n* = 6) and TXNDC12 overexpression with shc-Myc (*n* = 6). **h** Immunohistochemical analysis of Ki-67 staining intensities in xenograft tumors from each treatment group (scale bar, 100 μm).

Given that TXNDC12 enhances c-Myc expression at the post-transcriptional level, we investigated whether TXNDC12’s tumor-promoting role in HNSCC cells is primarily mediated by c-Myc. MTT, EdU and colony formation assays consistently demonstrated that depletion of c-Myc significantly suppressed the enhanced proliferative and colony-forming capabilities of cells overexpressing TXNDC12 (Fig. 5a–d). Similarly, the transwell invasion assay showed that the increased invasive capacity of HNSCC cells transduced with TXNDC12-overexpressing lentiviruses was diminished following c-Myc depletion (Fig. 5e). In addition, tumor volume and weight analyses in xenograft models supported these findings. Tumors derived from HNSCC cells overexpressing TXNDC12 showed a significant reduction in both volume and weight following the depletion of c-Myc (Fig. 5f, g). Moreover, in tumors from HNSCC cells with enforced TXNDC12 expression, Ki-67 immunostaining revealed a marked decrease upon c-Myc depletion (Fig. 5h). These results underscore the pivotal role of c-Myc in mediating the tumorigenic effects driven by TXNDC12 in HNSCC.

### TXNDC12-mediated c-Myc stabilization through USP5-dependent deubiquitination

**Fig. 6:**
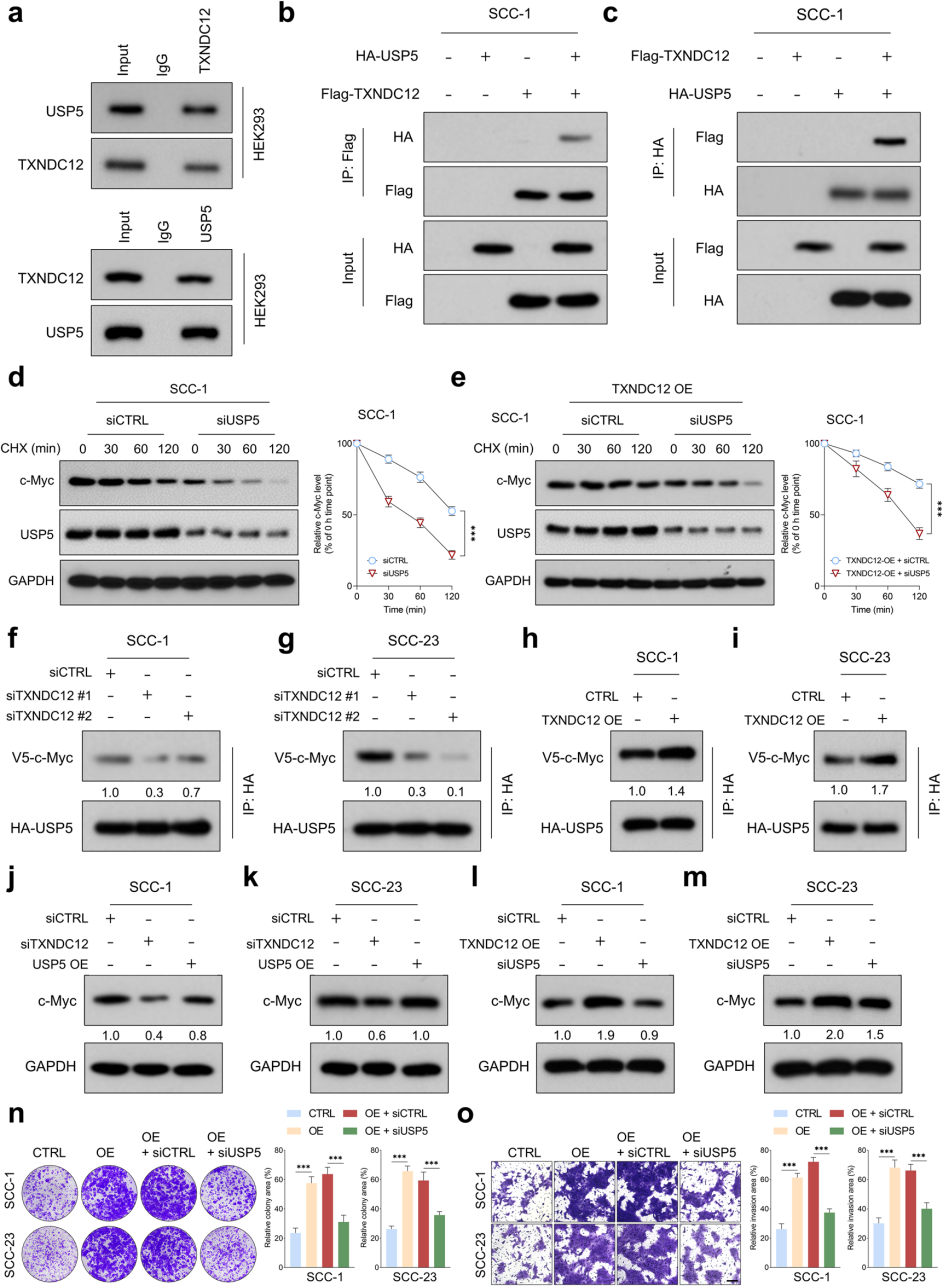
TXNDC12-mediated c-Myc stabilization through USP5-dependent deubiquitination. **a** Endogenous Co-IP analysis revealing the interaction between TXNDC12 and USP5 in HEK-293 cells (*n* = 3 biologically independent samples). **b**, **c** Exogenous Co-IP analysis demonstrating the interaction between Flag-TXNDC12 and HA-USP5 (**b**) or HA-USP5 and Flag-TXNDC12 (**c**) in HNSCC cells (*n* = 3 biologically independent samples). **d**, **e** CHX chase assays showing the effects of USP5 depletion on the degradation of c-Myc protein in SCC-1 cells, with (**d**) and without (**e**) TXNDC12 overexpression (*n* = 3 biologically independent samples). **f**, **g** Co-IP and western blot analyses assessing the impact of TXNDC12 depletion on the interaction between USP5 and c-Myc in SCC-1 (**f**) and SCC-23 cells (**g**) (*n* = 3 biologically independent samples). **h**, **i** Co-IP and western blot analyses examining the effect of TXNDC12 overexpression on the interaction between USP5 and c-Myc in SCC-1 (**h**) and SCC-23 (**i**) cells (*n* = 3 biologically independent samples). **j**–**m** A western blot analysis of c-Myc protein expression in HNSCC cells following the TXNDC12-depleted (**j, k**) or overexpressed (**l, m**) treatments (*n* = 3 biologically independent samples). **n**, **o** Assessment of colony formation (**n**) and invasive (**o**) capabilities of HNSCC cells under the specified treatments (scale bar, 200 μM) (*n* = 4 biologically independent samples).

To further explore the molecular mechanisms underlying TXNDC12-mediated c-Myc stabilization in HNSCC cells, we utilized coimmunoprecipitation (Co-IP) coupled with MS to identify proteins interacting with TXNDC12 (Supplementary Table 3). Among the top identified proteins, USP5, a deubiquitinating enzyme crucial for protein stability and function, was of particular interest. We hypothesized that TXNDC12 may stabilize c-Myc through its interaction with USP5. Both endogenous and exogenous Co-IP assays consistently demonstrated interactions between TXNDC12 and USP5 (Fig. 6a–c). As anticipated, CHX chase assays indicated that USP5 deletion significantly accelerated the degradation of c-Myc protein in HNSCC cells (Fig. 6d and Supplementary Fig. 9a). Notably, depletion of USP5 markedly reversed the enhanced stability of c-Myc protein conferred by TXNDC12 overexpression (Fig. 6e and Supplementary Fig. 9b). Co-IP further demonstrated that depletion of TXNDC12 suppressed the interaction between USP5 and c-Myc proteins, whereas TXNDC12 overexpression enhanced this interaction (Fig. 6f–i). These findings suggest that TXNDC12 mediates c-Myc stabilization through USP5-dependent deubiquitination.

We subsequently evaluated the role of USP5 in mediating the effects of TXNDC12 on c-Myc expression in HNSCC cells. The results demonstrated that overexpression of USP5 partially rescued the suppression of c-Myc protein levels caused by TXNDC12 depletion (Fig. 6j, k). Conversely, depletion of USP5 partially inhibited the increased expression of c-Myc protein facilitated by TXNDC12 overexpression (Fig. 6l, m). Both MTT and colony formation assays demonstrated that depleting USP5 significantly mitigated the enhanced proliferative and colony-forming activities conferred by overexpression of TXNDC12 (Fig. 6n and Supplementary Fig. 9c,d). Similarly, the Transwell invasion assay indicated that the reduction of USP5 curtailed the increased invasive potential facilitated by TXNDC12 overexpression (Fig. 6o). These observations suggest that USP5 is a key intermediary in the TXNDC12-c-Myc regulatory pathway in HNSCC.

### METTL1 promotes *TXNDC12* mRNA stability in an m^7^G‐dependent manner

To identify potential upstream regulators of TXNDC12, we examined genes that are both biologically relevant and consistently coexpressed with TXNDC12 across multiple independent HNSCC datasets. METTL1 was specifically selected for further investigation based on the following rationale: (1) it showed a strong and consistent positive correlation with TXNDC12 expression in several publicly available HNSCC cohorts; (2) this correlation was also observed in other tumor types, suggesting a broader regulatory association; and (3) METTL1 encodes an RNA methyltransferase known to influence mRNA stability and translation through epitranscriptomic mechanisms, which may functionally impact TXNDC12 expression at the post-transcriptional level.

**Fig. 7:**
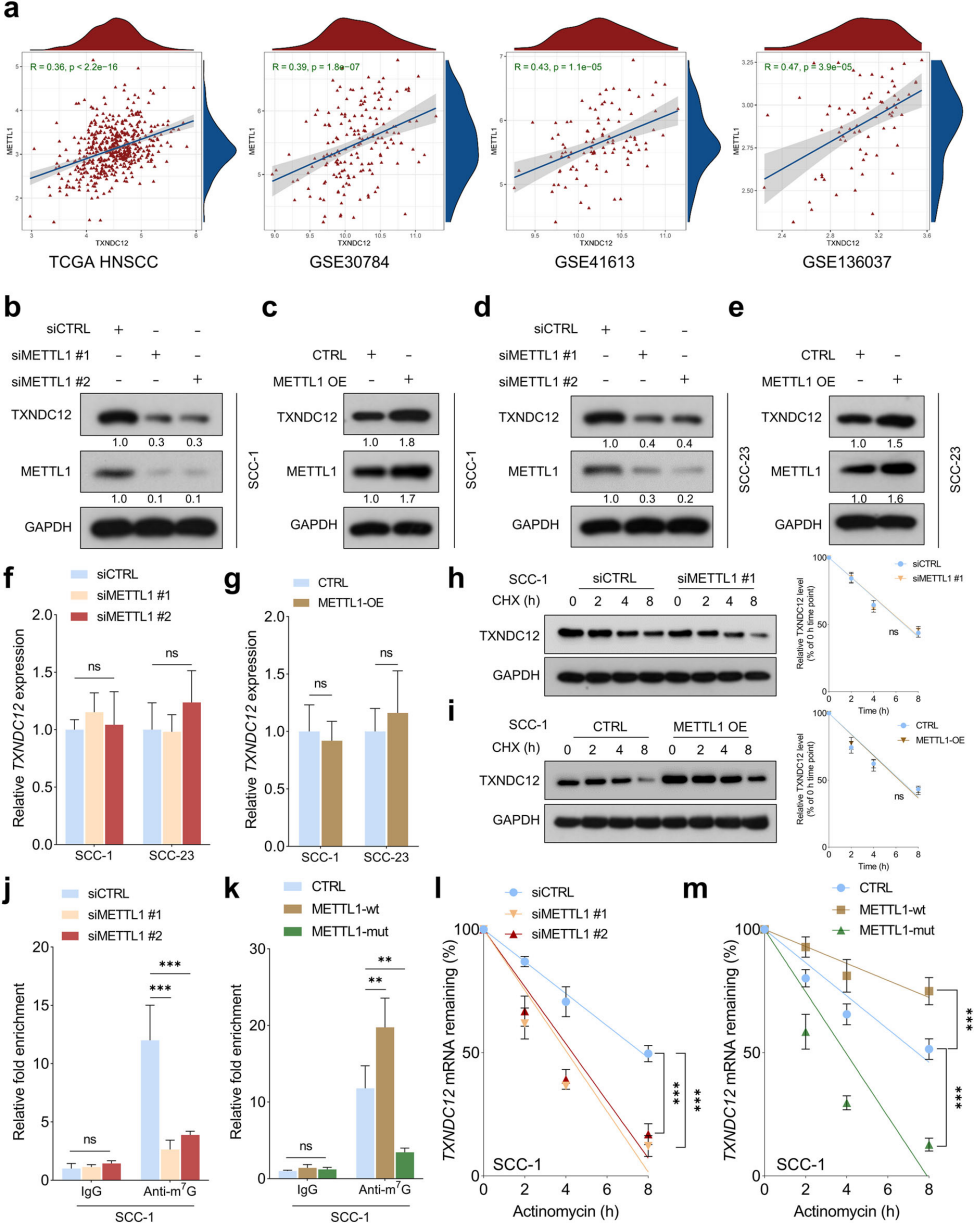
METTL1 promotes *TXNDC12* mRNA stability in an m^7^G‐dependent manner. **a** Correlation analysis between *METTL1* mRNA and *TXNDC12* mRNA across multiple HNSCC cohorts, including TCGA HNSCC, GSE30784, GSE41613 and GSE136037. **b**–**e** A western blot analysis of TXNDC12 protein levels in SCC-1 (**b, c**) or SCC-23 (**d, e**) cells with METTL1 depletion or overexpression, compared with their respective controls (*n* = 3 biologically independent samples). **f**, **g** A quantitative PCR analysis of *TXNDC12* mRNA levels following METTL1 depletion (**f**) or overexpression (**g**) in HNSCC cells (*n* = 3 biologically independent experiments, each with three technical replicates). **h**, **i** CHX chase assays demonstrating the effects of METTL1 depletion (**h**) or overexpression (**i**) on the degradation rate of TXNDC12 protein (*n* = 3 biologically independent samples). **j**, **k** The measurement of the internal m^7^G level of *TXNDC12* mRNA using m^7^G RNA immunoprecipitation with a m^7^G antibody, with METTL1 depletion (**j**) or METTL1 overexpression (**k**) treatments, including wild type (METTL1-wt) and mutant (METTL1-mut) forms (*n* = 3 biologically independent experiments, each with three technical replicates). **l**, **m** An analysis of remaining *TXNDC12* mRNA levels in actinomycin-D-treated SCC-1 cells at the indicated time points, with METTL1 depletion (**l**) or overexpression (**m**) treatments (*n* = 3 biologically independent experiments, each with three technical replicates).

Subsequent bioinformatic analysis confirmed a robust positive correlation between *METTL1* and *TXNDC12* expression across multiple independent HNSCC cohorts, including TCGA HNSCC, GSE30784, GSE41613, GSE136037, GSE2837, GSE25727, GSE40774 and GSE127165 (Fig. 7a and Supplementary Fig. 10). Notably, a similar correlation was also observed in other cancer types (Supplementary Fig. 11). Our results indicated that depletion of METTL1 markedly suppressed the expression of TXNDC12 protein, whereas overexpression of METTL1 enhanced its expression (Fig. 7b–e). However, quantitative PCR analysis revealed that changes in METTL1 levels, either by depletion or overexpression, did not significantly affect the mRNA levels of TXNDC12 in HNSCC cells (Fig. 7f,g). This suggests that METTL1 regulates TXNDC12 at the post-transcriptional level, independent of mRNA abundance. Further, CHX chase assays showed that these alterations in METTL1 did not significantly impact the degradation rate of TXNDC12 protein in HNSCC cells (Fig. 7h,i). Recent studies suggest that METTL1 plays a crucial role in m^7^G methylation of mRNA, thereby regulating mRNA export, splicing, translation efficiency and stability. Therefore, we hypothesize that METTL1 regulates *TXNDC12* mRNA stability in an m^7^G-dependent manner. The m^7^G RNA immunoprecipitation assay demonstrates that METTL1 knockdown significantly reduces the m^7^G modification of *TXNDC12* mRNA in HNSCC cells (Fig. 7j and Supplementary Fig. 12a). Conversely, overexpression of wild-type METTL1 significantly increases the m^7^G modification of *TXNDC12* mRNA, whereas the METTL1 mutant fails to enhance m^7^G enrichment (Fig. 7k and Supplementary Fig. 12b). Furthermore, actinomycin D treatment reveals that METTL1 knockdown accelerates the degradation of *TXNDC12* mRNA in HNSCC cells, with both siMETTL1-treated groups showing a more rapid decline in mRNA levels compared with the control siRNA (Fig. 7l and Supplementary Fig. 12c). Notably, following actinomycin D treatment, wild-type METTL1 overexpression significantly stabilizes *TXNDC12* mRNA, slowing its degradation rate, while the METTL1 mutant lacks this stabilizing effect (Fig. 7m and Supplementary Fig. 12d). These findings underscore the critical role of METTL1 in regulating the m^7^G modification and stability of *TXNDC12* mRNA in HNSCC cells.

### The clinical significance of METTL1-TXNDC12-c-Myc axis in HNSCC

**Fig. 8:**
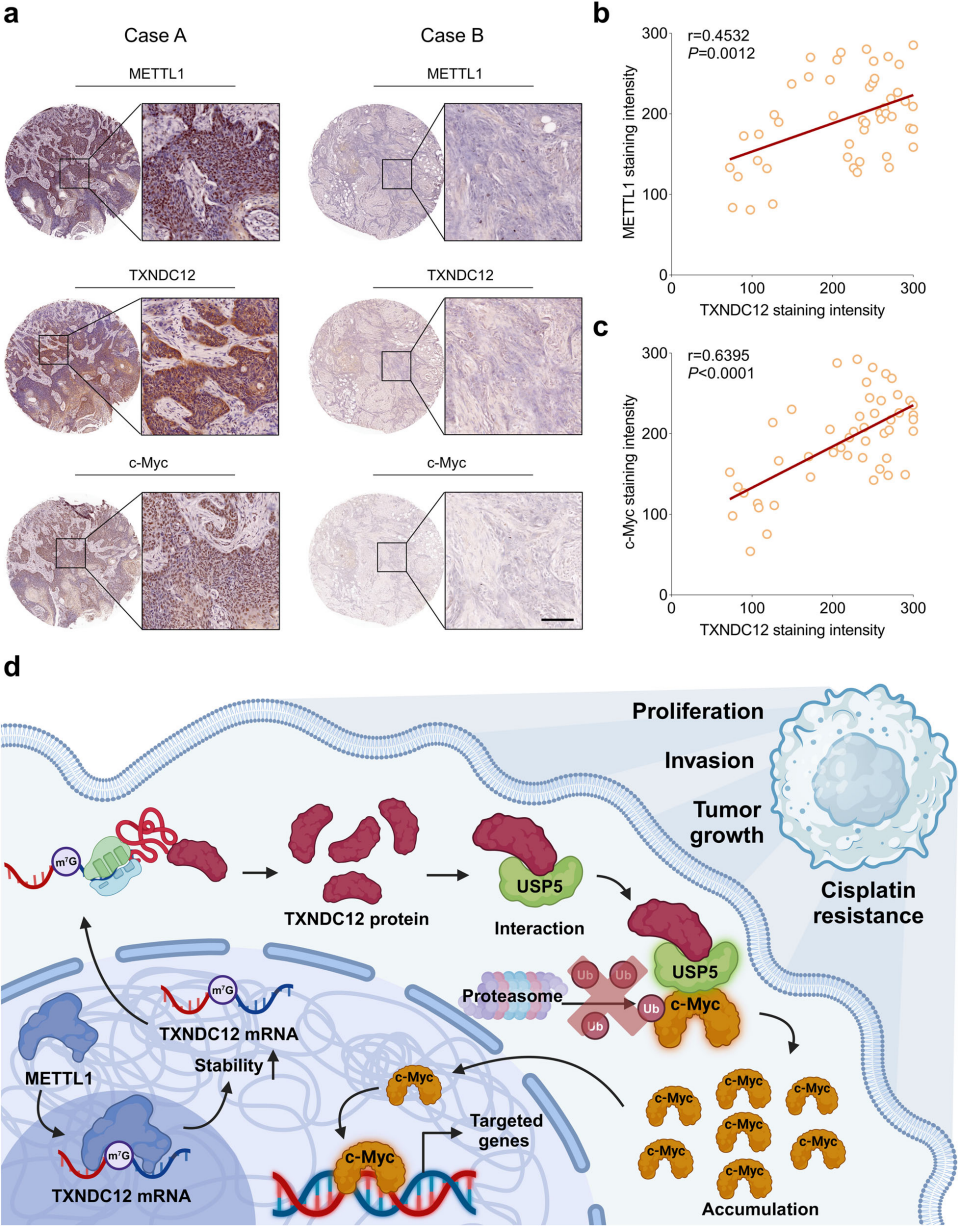
The clinical significance of METTL1-TXNDC12-c-Myc axis in HNSCC. **a** Representative images showing the expression levels of METTL1, TXNDC12 and c-Myc in two cases of HNSCC (scale bar, 100 μm). **b** A correlation analysis of METTL1 and TXNDC12 expression levels in HNSCC specimens (*n* = 48). **c** An analysis of the correlation between TXNDC12 and c-Myc expression levels in HNSCC specimens (*n* = 48). **d** A schematic diagram illustrating the mechanism by which TXNDC12 promotes HNSCC progression.

To assess the clinical relevance of the METTL1-TXNDC12-c-Myc axis in HNSCC, we performed immunohistochemical analyses on METTL1, TXNDC12 and c-Myc within an in-house HNSCC cohort. A quantitative analysis of staining intensities revealed a robust positive correlation between METTL1 and TXNDC12 protein levels (*r* = 0.4532, *P* = 0.0012) and, similarly, a strong correlation between TXNDC12 and c-Myc expression (*r* = 0.6395, *P* < 0.0001) (Fig. 8a–c). These findings indicate that higher levels of METTL1 are associated with increased TXNDC12 expression, which in turn correlates with elevated c-Myc levels, suggesting a sequential regulatory cascade. This molecular axis might contribute significantly to the oncogenic phenotype in HNSCC cells, underscoring potential targets for therapeutic intervention.

## Discussion

Our study reveals pronounced overexpression of TXNDC12 in HNSCC, associated with enhanced tumor aggressiveness and poor prognosis. TXNDC12 stabilizes c-Myc by augmenting its interaction with USP5, thus preventing c-Myc degradation via the proteasome. Moreover, METTL1 enhances *TXNDC12* mRNA stability through m^7^G methylation, elevating TXNDC12 levels and contributing to HNSCC progression. These findings delineate the regulatory role of the METTL1-TXNDC12-c-Myc axis in the pathogenesis of HNSCC, as detailed in Fig. 8d.

The TXNDC family, characterized by its thioredoxin domains, plays a crucial role in regulating protein folding and signaling pathways that are vital for cellular function and survival^20,21^. Our clinical and experimental evidence consistently demonstrates that TXNDC12 is a critical driver of HNSCC progression. HNSCC remains a significant therapeutic challenge, largely due to the absence of effective molecular markers that can guide treatment decisions and predict outcomes^22^. The identification of TXNDC12 as a novel molecular target is particularly promising. Our findings indicate that TXNDC12 not only promotes tumor aggressiveness but also plays a role in modulating the cellular response to cisplatin. Specifically, we demonstrate that depletion of TXNDC12 significantly increases the sensitivity of HNSCC cells to cisplatin, whereas its overexpression induces a more resistant phenotype. These results suggest that TXNDC12 may serve as a critical regulator of cisplatin responsiveness and a potential therapeutic target for improving treatment outcomes in HNSCC. Future research should focus on developing specific inhibitors of TXNDC12 or employing nanotechnology-based delivery systems, such as liposomes, for targeted delivery of TXNDC12 siRNA. These approaches may provide new opportunities to optimize cisplatin-based treatment and improve clinical outcomes in HNSCC.

Through comprehensive RNA transcriptomic analyses, bioinformatics assessments of publicly accessible datasets and functional rescue experiments, we have demonstrated that TXNDC12 promotes tumorigenesis in HNSCC by activating the c-Myc signaling pathway, primarily through enhancing its stability. c-Myc, a pivotal oncogene, regulates cellular proliferation, metabolism and apoptosis and is associated with aggressive cancer phenotypes and poor prognosis^23,24^. It also plays a significant role in chemotherapy resistance, notably to cisplatin, by altering DNA repair mechanisms and enhancing cellular survival pathways, thereby enabling cancer cells to evade drug-induced apoptosis^25,26^. Given that TXNDC12’s tumor-promoting effects are largely mediated through c-Myc, it is logical that depleting TXNDC12 leads to profound changes in tumor cell phenotypes both in vitro and in vivo. Considering c-Myc’s status as ‘difficult-to-drug’ or ‘undruggable’^27^, our findings highlight the targeting of TXNDC12 as a novel and effective approach to circumvent the challenges posed by c-Myc in cancer therapy, underscoring the importance of this strategy in advancing cancer treatment.

Mechanistically, we demonstrate that TXNDC12 stabilizes c-Myc by enhancing its interaction with USP5, thereby preventing proteasomal degradation of c-Myc. USP5 is a critical enzyme in the ubiquitin–proteasome system, playing a pivotal role in regulating cellular protein homeostasis^28^. This protease disassembles unanchored polyubiquitin chains, essential for recycling ubiquitin and maintaining its cellular pool^29^. Beyond its core functions, USP5 is increasingly recognized for its role in modulating signaling pathways crucial for tumor progression and drug resistance, making it a target of interest in cancer research^30^. For instance, USP5 is identified as a novel PD-L1 deubiquitinase in non-small cell lung cancer, enhancing PD-L1 protein stability and promoting immune escape^31^. Similarly, USP5 is identified as a novel deubiquitinase for HIF2α in breast cancer, interacting with and stabilizing HIF2α by preventing its ubiquitin–proteasome degradation. This stabilization promotes the transcription of HIF2α target genes, enhancing breast cancer cell proliferation, migration and invasion^32^. In addition, USP5 stabilizes Twist1 through its deubiquitinase activity, activating the epithelial–mesenchymal transition pathway in bladder cancer. Depletion of USP5 reduces proliferation, invasion and epithelial–mesenchymal transition, whereas elevated USP5 levels correlate with increased Twist1 in clinical samples. Targeting the USP5-Twist1 axis offers a potential therapeutic strategy for bladder cancer^33^.

Importantly, we further demonstrate that METTL1 enhances *TXNDC12* mRNA stability through m^7^G-dependent mechanisms. METTL1 is a well-characterized methyltransferase primarily known for its role in catalyzing the m^7^G modification in tRNA, which is crucial for maintaining tRNA stability and function^34^. This modification plays a significant role in the progression of various cancers^35,36^. For instance, depletion of METTL1/WDR4 impairs m^7^G tRNA modification, reducing cell proliferation, invasion and tumorigenicity^37^. METTL1 enhances lung cancer growth and invasion by regulating m^7^G tRNA modifications, affecting mRNA translation of codon-biased transcripts, thus driving cancer progression^38^. Similarly, METTL1 catalyzes m^7^G modification of tRNAs, notably Arg-TCT-4-1, enhancing the translation of mRNAs, including cell cycle regulators. METTL1 depletion disrupts cell cycle and oncogenicity, while its overexpression induces oncogenic transformation^39^. Recent studies have expanded the scope of METTL1’s functions, revealing its ability to modify mRNA, thereby promoting tumor growth through enhanced mRNA stability and translation^40^. In our research, we have identified *TXNDC12* as a novel epigenetic target of METTL1 in HNSCC. We demonstrate that METTL1 promotes the stability of *TXNDC12* mRNA in an m^7^G-dependent manner, thereby facilitating its role in HNSCC tumorigenesis. This finding elucidates a new dimension of METTL1’s contribution to cancer progression, highlighting TXNDC12 as a pivotal player in HNSCC and a potential therapeutic target.

In summary, our findings reveal that METTL1-driven epitranscriptomic upregulation of TXNDC12 in HNSCC enhances c-Myc signaling by promoting its USP5-mediated stability. This study elucidates the complex molecular mechanisms underlying HNSCC tumorigenesis and highlights TXNDC12 as a promising therapeutic target.

## Supplementary information

Supplementary Information

## Data Availability

The data that support the findings of this study are available from the corresponding authors upon reasonable request.
