# Supplementary material for: METTL1-driven epitranscriptomic enhancement of TXNDC12 boosts c-Myc stability through USP5 in HNSCC
Source: Exp Mol Med. 2025 Aug 1;57(8):1743–58. doi: 10.1038/s12276-025-01512-2 (PMC12411639; doi:10.1038/s12276-025-01512-2)
Supplement: Supplementary file 1 — Supplementary Information [file 12276_2025_1512_MOESM1_ESM.pdf]

METTL1-Driven Epitranscriptomic Enhancement of TXNDC12 Boosts c-Myc Stability Through USP5 in HNSCC

Zizhao Mai[1, *], Jiarong Zheng[2, *], Ye Lu[1], Pei Lin[1], Yunfan Lin[1], Yucheng Zheng[1], Xu Chen[1], Bing Guo[2], Li Cui,[1,3, #] Xinyuan Zhao[1, #]

[1] Stomatological Hospital, School of Stomatology, Southern Medical University, Guangzhou 510280, Guangdong, China.

[2] Department of Dentistry, The First Affiliated Hospital, Sun Yat-Sen University, Guangzhou 510080, Guangdong, China.

[3] School of Dentistry, University of California, Los Angeles, Los Angeles, 90095, CA, USA.

*These authors contributed equally to this work

[#]Please address the correspondence to:

Li Cui, Email: licui@smu.edu.cn

Xinyuan Zhao, Email: zhaoxinyuan1989@smu.edu.cn

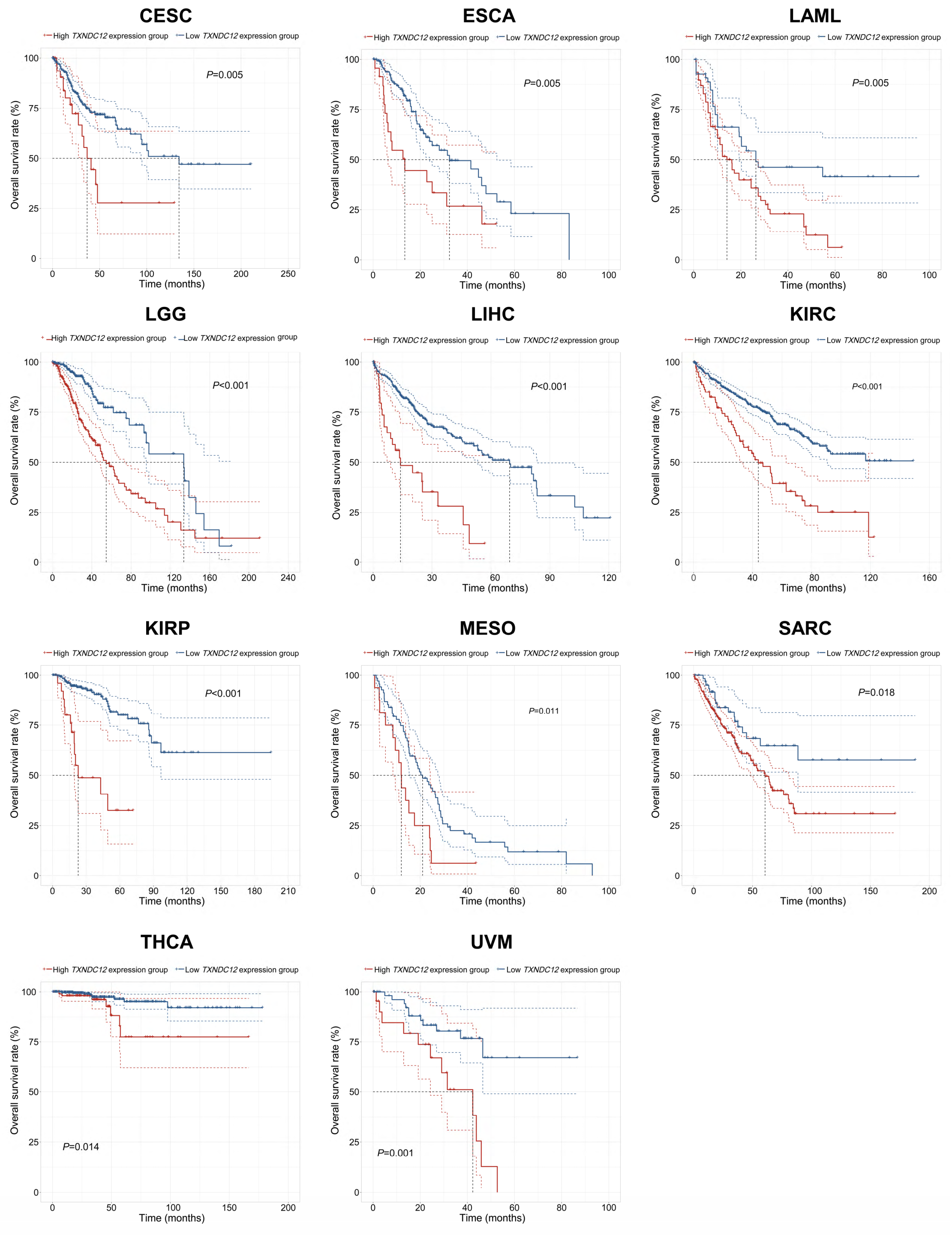

Supplementary Figure 1. Survival analysis stratifying cancer patients by *TXNDC12* expression across TCGA cohorts, including CESC, ESCA, LAML, LGG, LIHC, KIRC, KIRP, MESO, SARC, THCA, and UVM. Statistical significance was calculated using the log-rank test.

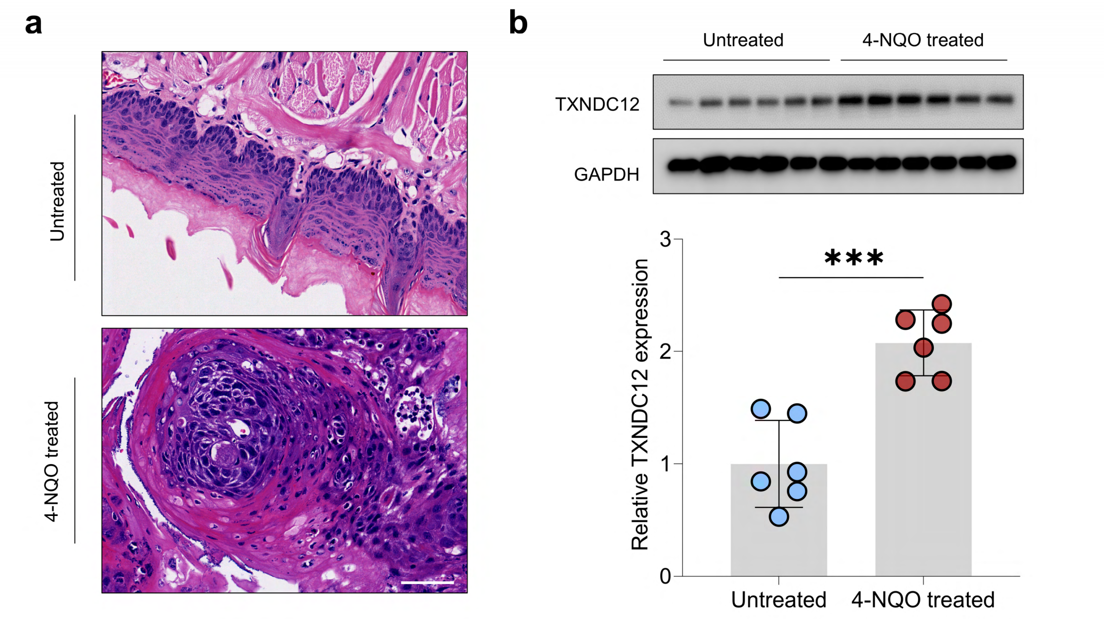

Supplementary Figure 2. TXNDC12 is overexpressed in cancer tissues compared to normal tongue tissues in the 4-NQO-induced HNSCC mouse model. (a) Representative HE images of normal tongue and carcinogenesis tissues induced by 4-NQO (Scale bar=50 μM). (b) Western blot analysis of TXNDC12 expression in untreated (n=6) and 4-NQO-treated tumor tissues (n=6).

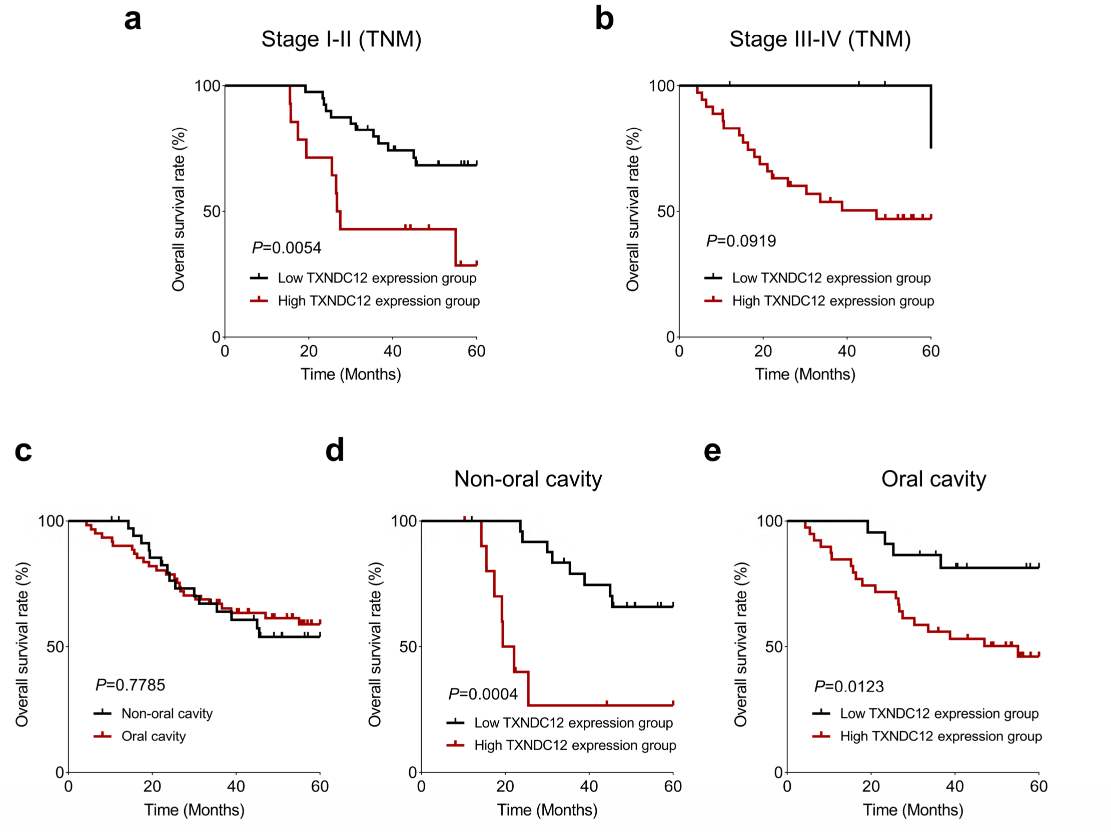

Supplementary Figure 3. TXNDC12 expression is associated with poor prognosis in defined subgroups of the in-house HNSCC cohort. (a-b) Kaplan-Meier survival analyses comparing overall survival between high and low TXNDC12 expression groups in patients with TNM early-stage and advanced-stage HNSCC. (c) Comparison of overall survival between oral cavity and non-oral cavity tumors, independent of TXNDC12 expression. (d-e) Stratified survival analyses evaluating the prognostic impact of TXNDC12 expression within non-oral cavity and oral cavity tumor subgroups. Statistical significance was calculated using the log-rank test.

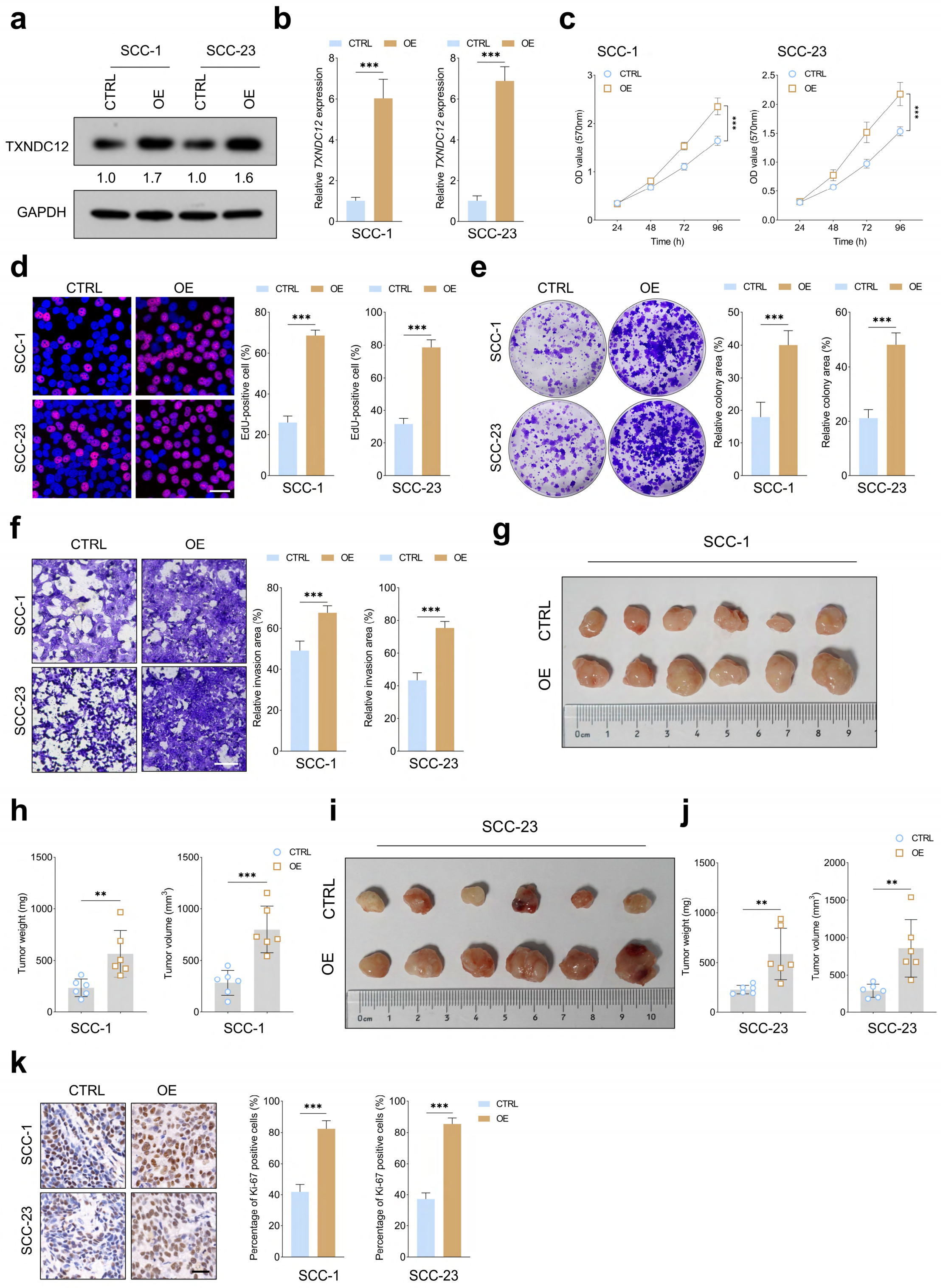

Supplementary Figure 4. TXNDC12 overexpression enhances the malignant characteristics of HNSCC cells. (a-b) Western blot (n = 3 biologically independent samples) and quantitative PCR analyses (n=3 biologically independent experiments, each with three technical replicates) were performed to evaluate TXNDC12 expression in HNSCC cells transfected with TXNDC12-overexpressing lentiviruses or control lentiviruses. (c-e) MTT (n=3 biologically independent experiments, each with five

technical replicates), EdU (n = 4 biologically independent samples), and colony formation assays (n = 4 biologically independent samples) were utilized to assess the proliferative and clonogenic potentials of TXNDC12-overexpressing cancer cells compared to controls (Scale bar=50 μm). (f) Invasion assays were conducted to examine the differences in invasive behaviors between TXNDC12-overexpressing cancer cells and their control counterparts (Scale bar=200 μm) (n = 4 biologically independent samples). (g-j) In the nude mouse subcutaneous model, quantitative assessments of tumor growth were conducted, comparing the volume and weight of tumors from the TXNDC12-overexpressing group (n=6) to those from the control group (n=6). (k) Immunohistochemical evaluation of Ki-67 staining intensities in xenograft tumors originating from TXNDC12-overexpressing cells and control cells (Scale bar=100 μm).

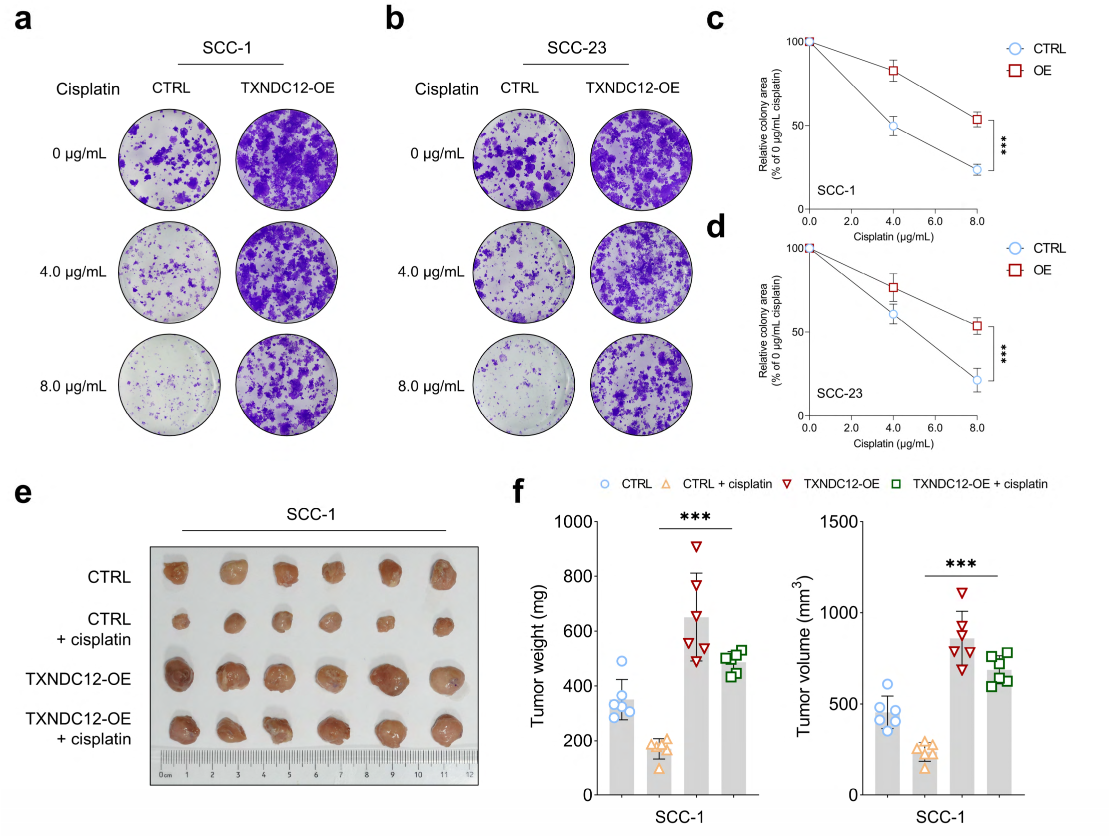

Supplementary Figure 5. In vitro and in vivo evaluation of the effects of TXNDC12 overexpression on cisplatin treatment in HNSCC cells. (a-d) Colony formation assays were performed in SCC-1 and SCC-23 cells transduced with control or TXNDC12-overexpressing lentiviruses and treated with cisplatin at the indicated concentrations. (e-f) In a nude mouse subcutaneous model, SCC-1 (1×10^6 cells per mouse) were injected subcutaneously to establish tumors. Tumor growth was quantitatively evaluated by comparing tumor volume and weight across four groups: CTRL (n=6), CTRL + cisplatin-treated (5 mg/kg, intraperitoneally every 5 days) (n=6), TXNDC12-OE (n=6), and combined cisplatin + TXNDC12-OE (n=6).

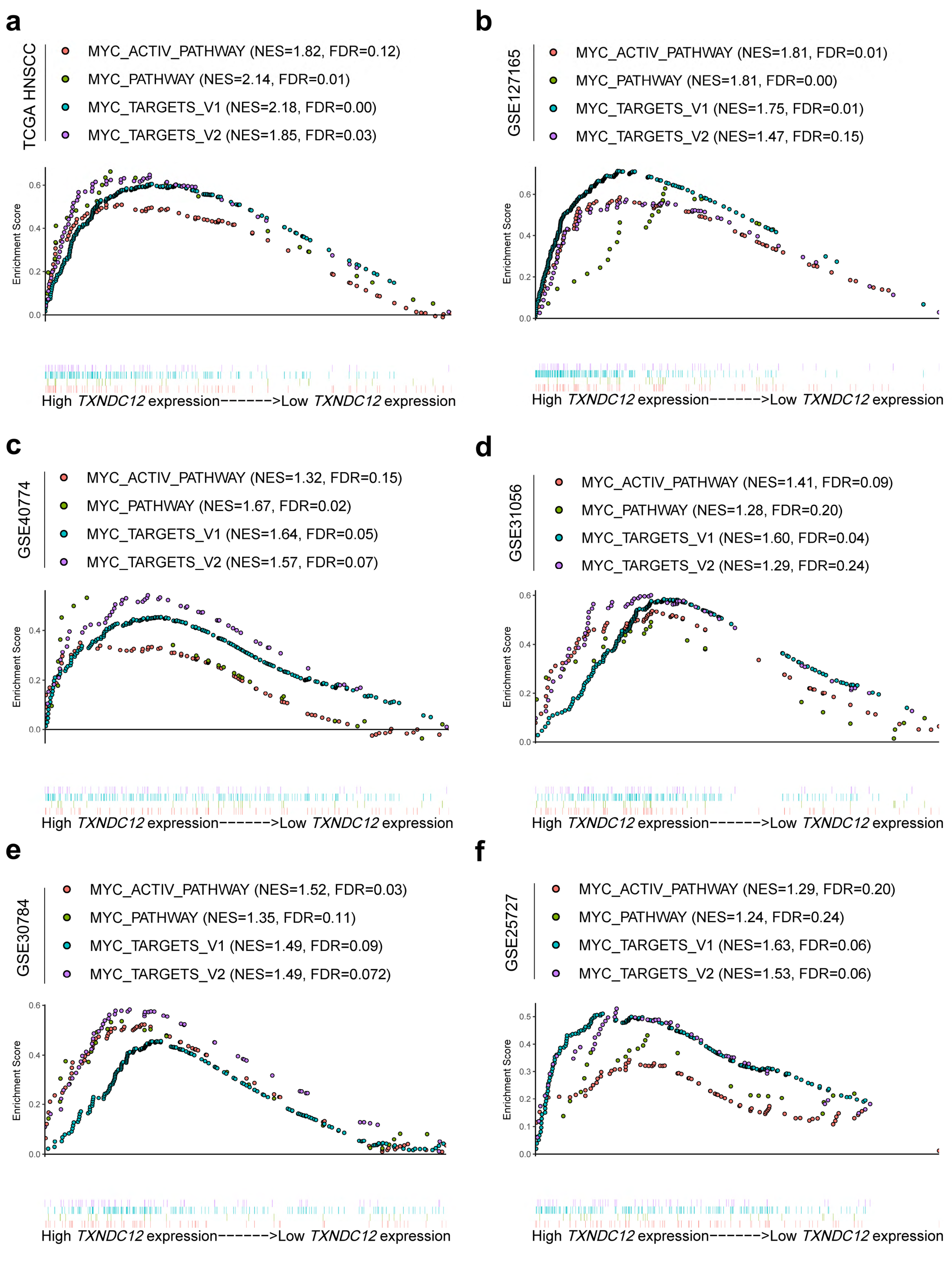

Supplementary Figure 6. GSEA analysis showing the enrichment of c-Myc-related signatures in the high *TXNDC12* expression group across various HNSCC cohorts. (a) Enrichment of c-Myc-related signatures within the TCGA HNSCC cohort. (b-f) Analysis of GEO datasets (GSE127165, GSE40774, GSE31056, GSE30784, and GSE25727) highlighting the enrichment of c-Myc-related signatures.

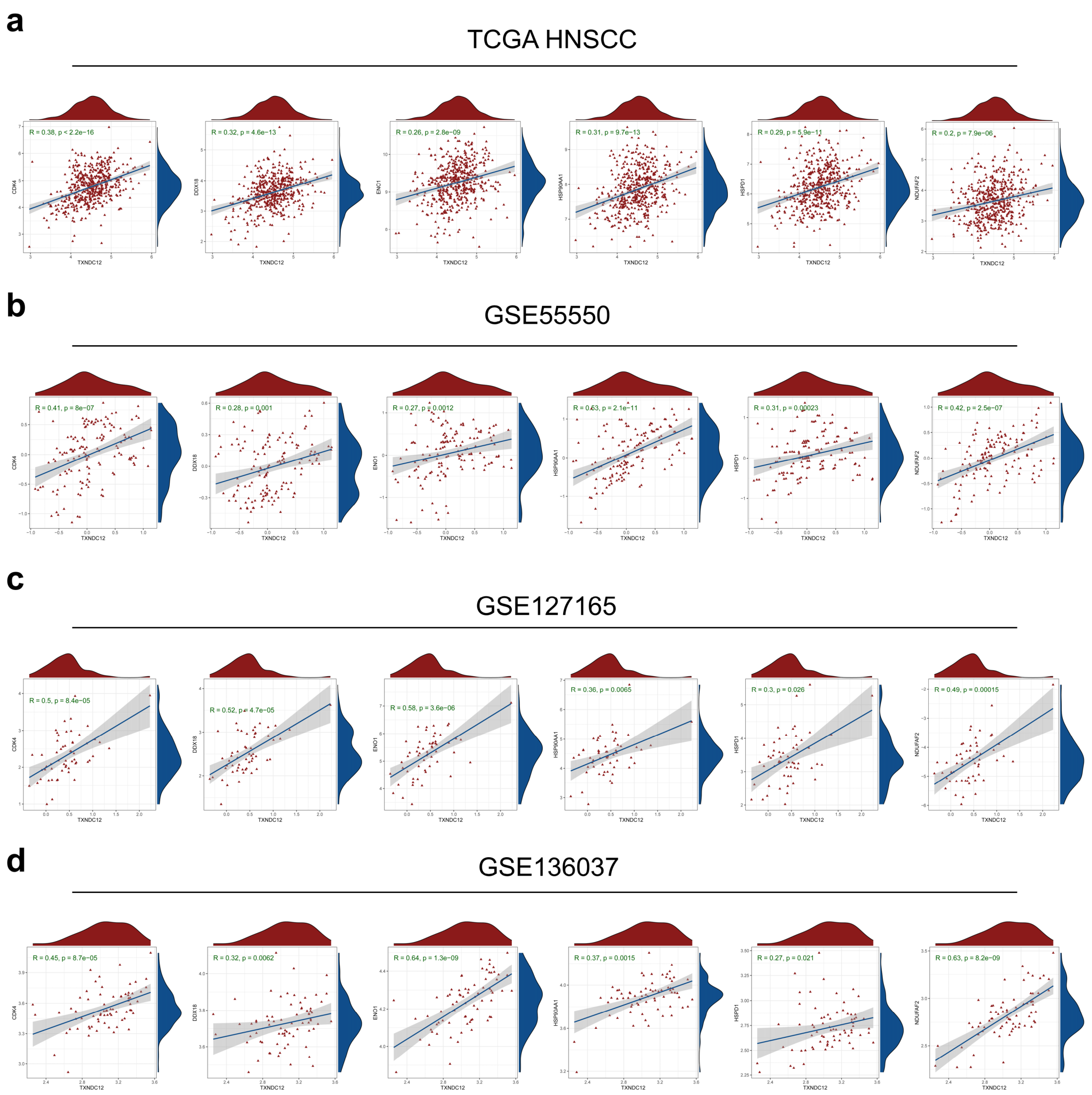

Supplementary Figure 7. Correlation analysis between *TXNDC12* mRNA and c-Myc downstream target genes across various HNSCC cohorts. (a-d) Correlation analysis between *TXNDC12* mRNA and c-Myc target genes (*CDK4*, *DDX18*, *ENO1*, *HSP90AA1*, *HSPD1*, and *NDUFAF2*) in TCGA HNSCC, GSE55550, GSE127165, and GSE136037 datasets.

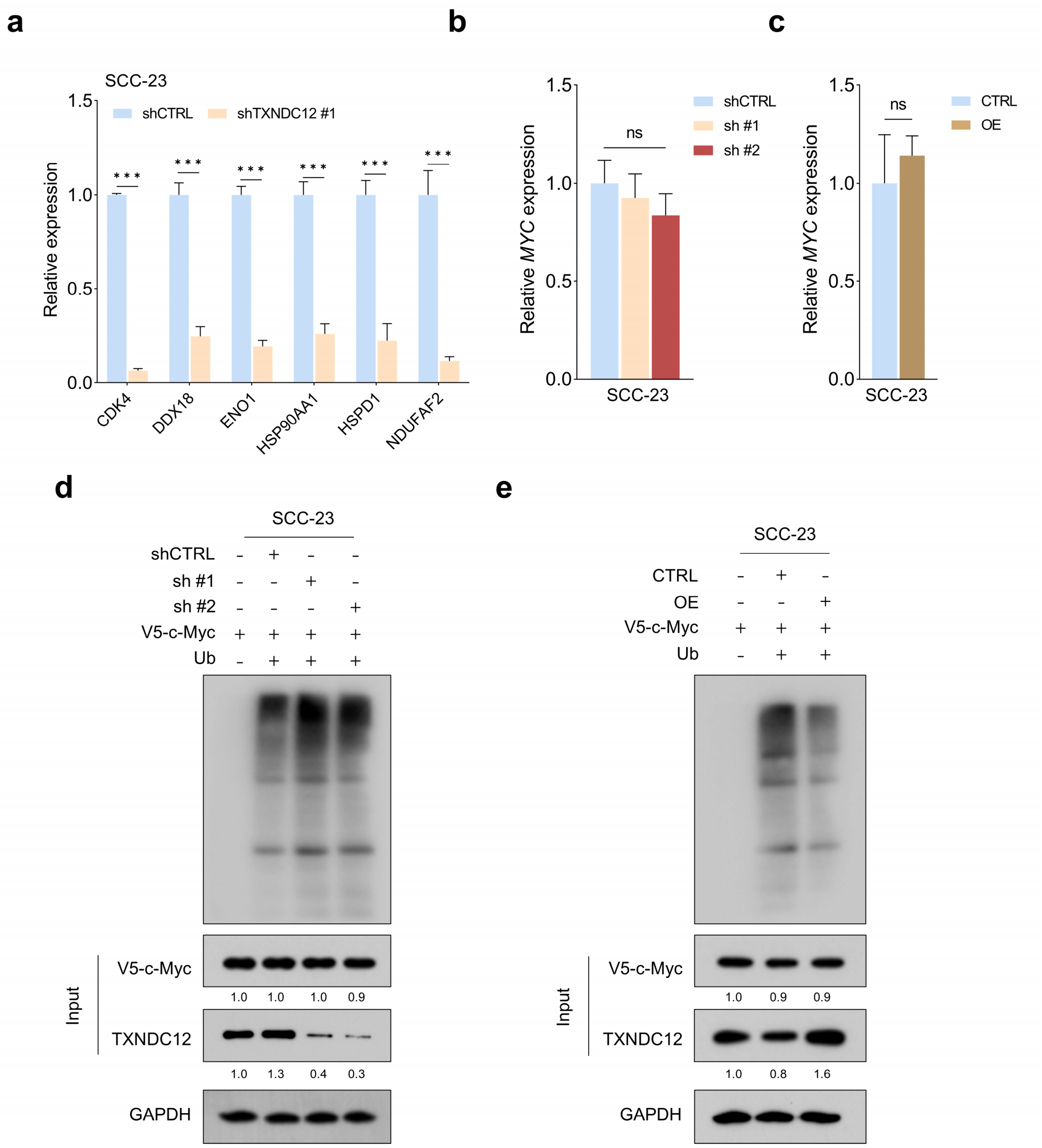

Supplementary Figure 8. TXNDC12 plays a crucial role in maintaining c-Myc protein stability in HNSCC cells. (a) Quantitative PCR analysis demonstrating the effect of TXNDC12 depletion on the expression of c-Myc target genes in SCC-23 cells (n=3 biologically independent experiments, each with three technical replicates). (b-c) Quantitative PCR results showing the impact of TXNDC12 depletion or overexpression on *MYC* mRNA levels in SCC-23 cells (n=3 biologically independent experiments, each with three technical replicates). (d-e) Analysis of ubiquitinated c-Myc expression in SCC-23 cells following the indicated treatments (n =3 biologically independent samples).

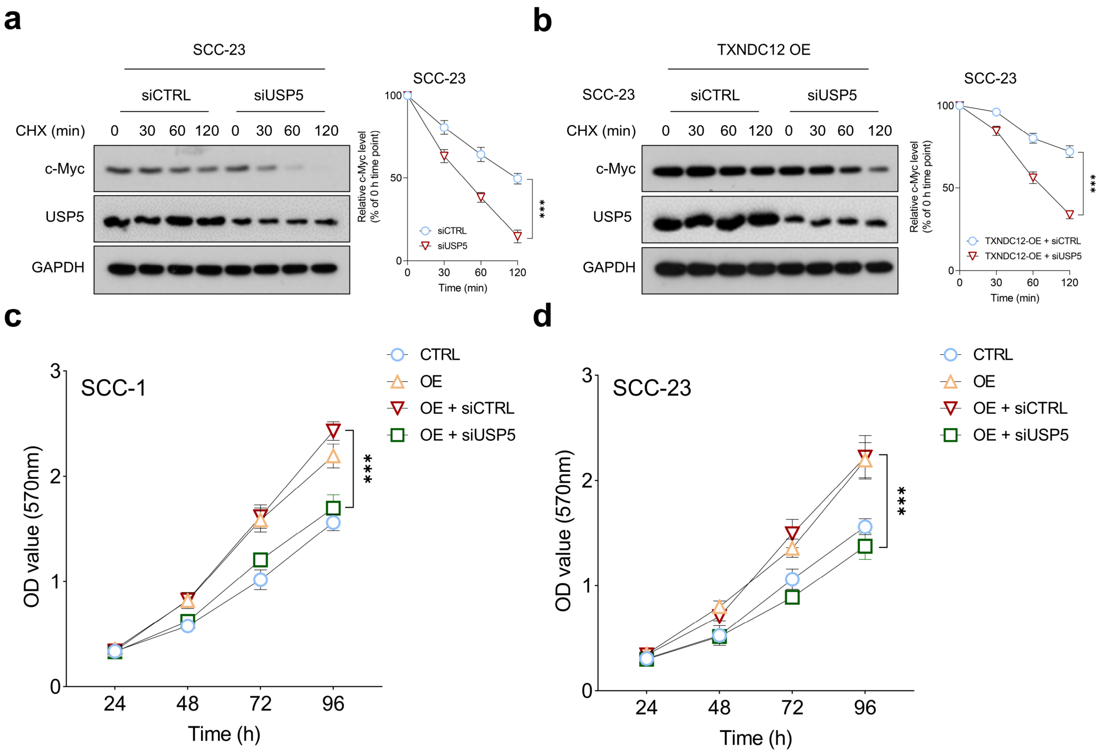

Supplementary Figure 9. TXNDC12-mediated stabilization of c-Myc via USP5-dependent deubiquitination. (a-b) CHX chase assays illustrating the impact of USP5 depletion on c-Myc protein degradation in SCC-23 cells, both in the presence and absence of TXNDC12 overexpression (n =3 biologically independent samples). (c-d) MTT assays assessing the proliferative capacity of HNSCC cells under the specified treatments (n=3 biologically independent experiments, each with five technical replicates).

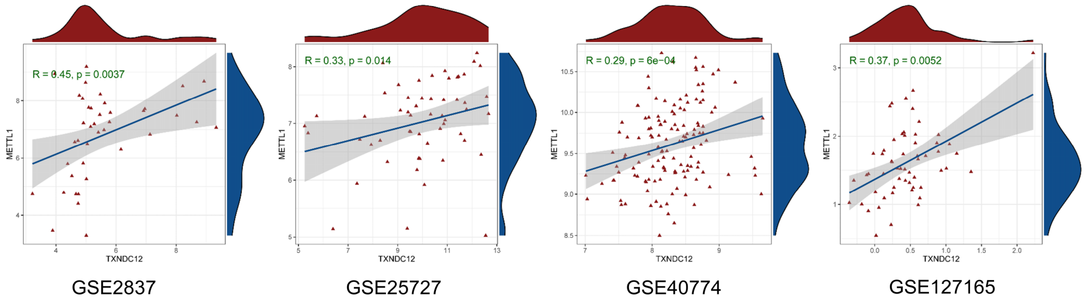

Supplementary Figure 10. Correlation analysis between *METTL1* and *TXNDC12* mRNA levels across several HNSCC cohorts, including GSE2837, GSE25727, GSE40774, and GSE127165.

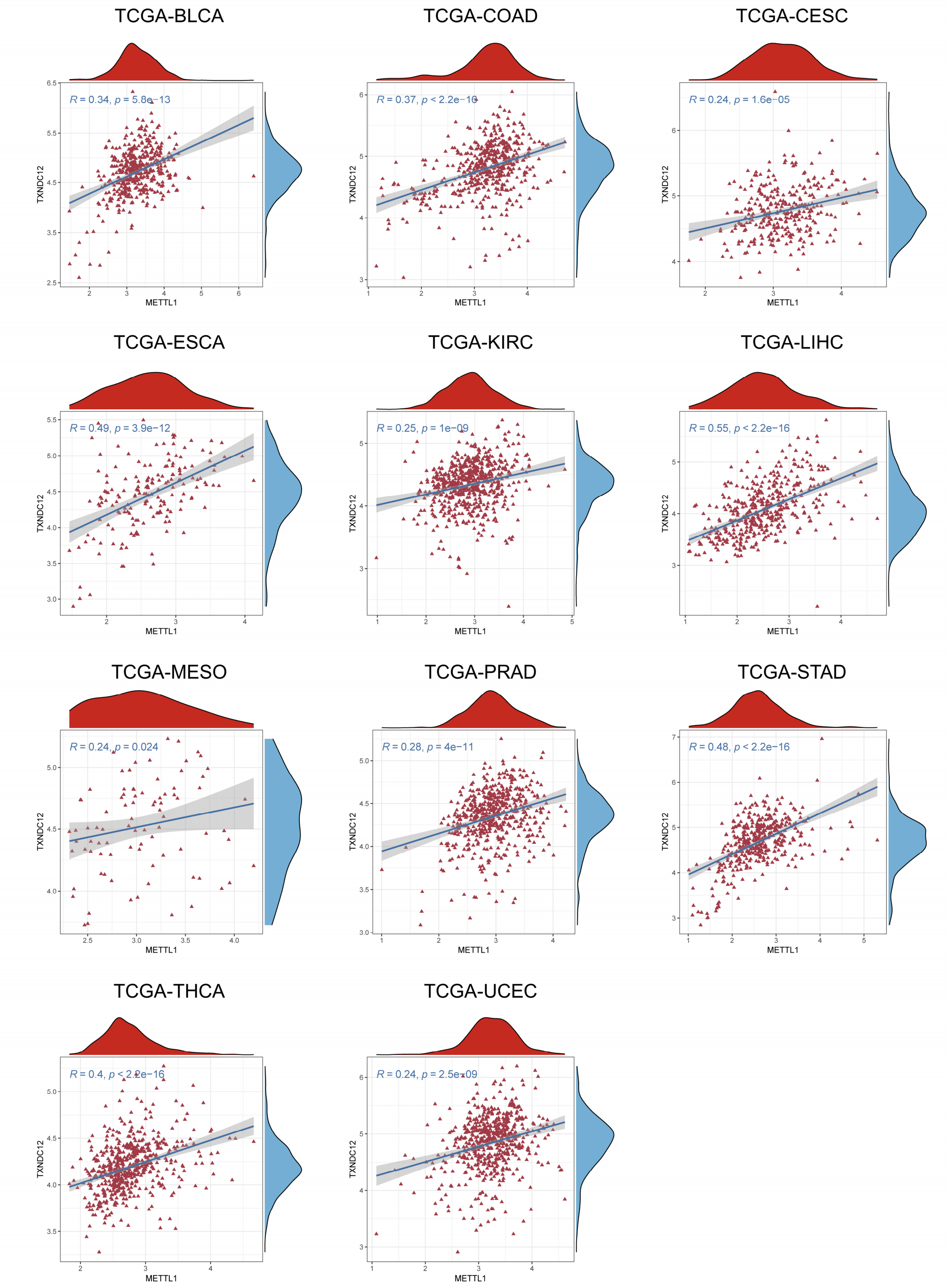

Supplementary Figure 11. Correlation analysis between *METTL1* and *TXNDC12* mRNA levels in TCGA-BLCA, TCGA-COAD, TCGA-CESC, TCGA-ESCA, TCGA-KIRC, TCGA-LIHC, TCGA-MESO, TCGA-PRAD, TCGA-STAD, TCGA-THCA and TCGA-UCEC.

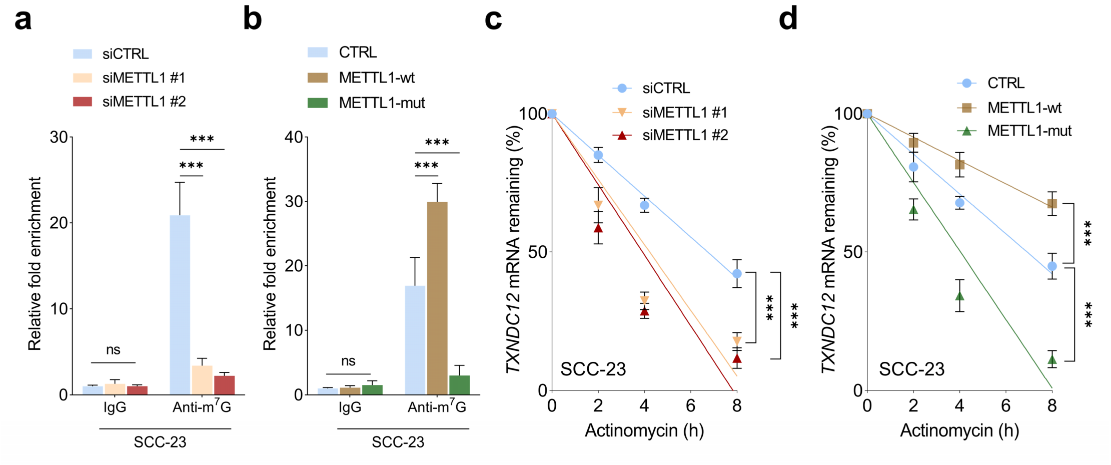

Supplementary Figure 12. METTL1 enhances the stability of TXNDC12 *mRNA* via m^{7}G-dependent mechanisms. (a-b) Quantification of the internal m^{7}G modification of *TXNDC12* mRNA in SCC-23 cells using m^{7}G RNA immunoprecipitation with a m^{7}G specific antibody under various treatment conditions (n=3 biologically independent experiments, each with three technical replicates). (c-d) Evaluation of *TXNDC12* mRNA stability in actinomycin D-treated SCC-23 cells, indicating the remaining mRNA levels at specified time points (n=3 biologically independent experiments, each with three technical replicates).

Supplementary Table 1. Sequences of primers and oligos used in this study.

Oligos	Sequence (5′-3′)
TXNDC12	F: GGACATAATGGGCTTGGAAAGGG
	R: CTTTGCAAGCTCCACACCAGGA
GAPDH	F: TGCACCACCAACTGCTTAGC
	R: GGCATGGACTGTGGTCATGAG
CDK4	F: CCATCAGCACAGTTCGTGAGGT
	R: TCAGTTCGGGATGTGGCACAGA
DDX18	F: GATGTGGCAGCGAGAGGACTAG
	R: GGCGCAAAATGAGCAAGGCATG
ENO1	F: AGTCAACCAGATTGGCTCCGTG
	R: CACAACCAGGTCAGCGATGAAG
HSP90AA1	F: TCTGCCTCTGGTGATGAGATGG
	R: CGTTCCACAAAGGCTGAGTTAGC
HSPD1	F: TGCCAATGCTCACCGTAAGCCT
	R: AGCCTTGACTGCCACAACCTGA
NDUFAF2	F: AAGGGAAGTGAAGGAGCACG
	R: CCAAGCTTCCCATTCTGTTGG
shTXNDC12 #1	CCTGATGGTGATTATTCATAA
shTXNDC12 #2	GCCTTCAGAAAGAAACATCTT
siUSP5	GACCACACGATTTGCCTCATT
siMETTL1	CCCACATTTCAAGCGGACAAA

Supplementary Table 2. Association of TXNDC12 expression with clinicopathological features in HNSCC.

Clinical parameters	TXNDC12 expression		*P*
	Low	High	
**Age**			0.830
≥60	33	32	
<60	15	17	
**Gender**			0.785
Male	40	42	
Female	8	7	
**Smoking status**			0.419
Yes	21	26	
No	27	23	
**Differentiation**			<0.001
G1-G2	42	24	
G3	6	25	
**TNM stage**			<0.001
I-II	40	14	
III-IV	8	35	
**Lymph node metastasis**			<0.001
Yes	5	24	
No	43	25	

Supplementary Table 3. The proteins interacting with TXNDC12 identified by mass spectrometry

UniProt No.	Gene symbol	Protein name	Protein score	Unique_Peptide_Num
Q14204	DYNC1H1	Cytoplasmic dynein 1 heavy chain 1	381.16	105
P21333	FLNA	Filamin-A	329.55	77
Q13813	SPTAN1	Spectrin alpha chain, non-erythrocytic 1	276.12	74
P78527	PRKDC	DNA-dependent protein kinase catalytic subunit	263.91	77
P45974	USP5	Ubiquitin carboxyl-terminal hydrolase 5	255.31	64
Q00610	CLTC	Clathrin heavy chain 1	221.53	55
Q01082	SPTBN1	Spectrin beta chain, non-erythrocytic 1	193.43	52
P10809	HSPD1	60 kDa heat shock protein, mitochondrial	168.25	43
P13667	PDIA4	Protein disulfide-isomerase A4	159.81	41
P14625	HSP90B1	Endoplasmin	157.08	37
